# The productive processing of formulaic sequences by second language learners in writing

**DOI:** 10.3389/fpsyg.2024.1281926

**Published:** 2024-02-21

**Authors:** Kunmeng Fan, Haixiao Wang

**Affiliations:** ^1^School of Foreign Studies, Nanjing University, Nanjing, China; ^2^School of Foreign Languages, Anhui University of Technology, Ma’anshan, China; ^3^Department of Applied Foreign Language Studies, Nanjing University, Nanjing, China

**Keywords:** formulaic sequence, productive processing, holistic processing, learner-internal formulaic sequence, language proficiency, topic familiarity

## Abstract

There has been much debate in psycholinguistic research on whether formulaic sequences (FSs) are processed holistically or in a compositional manner. Whereas most previous studies on this issue focused on the receptive processing of FSs, few have investigated the productive processing of FSs, particularly in the second language (L2) learning context. Besides, most previous studies on L2 FSs examined learner-external FSs, or those identified by external criteria such as corpus frequency with little attention to learner-internal FSs, or psychological units perceived as wholes by learners themselves, although there might be much overlap between learner-external and learner-internal FSs. This study was designed to explore the productive processing of FSs by L2 learners from their own perspective, while taking into account the effects of L2 proficiency and topic familiarity. It made a distinction between internal FSs and purely external FSs as the primary criterion of categorizing learners’ processing behaviors. Ten Chinese English learners from two proficiency levels completed two writing tasks differing in topic familiarity. Upon the completion of each task, each participant and the researcher identified the FSs separately and then distinguished internal FSs and purely external FSs (termed as assembled FSs, since they were perceived as being assembled from scratch) collectively. Next, each participant performed video stimulated recall (VSR) for the production process of each FS. The results showed that the learners’ conscious processing (i.e., retrieval/assembly and integration into the text) of FSs can be categorized on two levels (lexical and syntactic). There was more holistic processing than compositional processing on the lexical level, but not on the syntactic level, indicating the learners’ sizable storages of FSs and the syntactic flexibility of FSs. Furthermore, between-group differences and between-task differences were detected on two processing levels: higher-proficiency students retrieved more internal FSs and made more modifications to them than their lower-proficiency counterparts; in the familiar-topic writing, learners retrieved more internal FSs and made less modifications to them. Based on the findings, a model of L2 FS production is proposed, and pedagogical implications for the teaching of L2 FSs are provided.

## 1 Introduction

Formulaic sequences (FSs), referring to conventionalized and recurrent word combinations such as idioms, collocations and lexical bundles, have attracted extensive attention from a variety of research fields. In recent years, there has been much debate in psycholinguistic research about how FSs are stored and processed by language users (Siyanova-Chanturia, 2015; Kessler et al., 2020). Some research concludes that FSs are processed holistically without the involvement of grammatical analysis, as they have been found to be processed faster than non-formulaic language (e.g., Underwood et al., 2004; Jiang and Nekrasova, 2007; Millar, 2011; Tremblay et al., 2011; Kim and Kim, 2012; Hallin and Van Lancker Sidtis, 2017). Nevertheless, a growing number of studies have demonstrated that FSs are stored with “live” internal syntactic structures and undergo the same regular syntactic analysis as non-formulaic language, thus suggesting (partial) compositionality of FSs (e.g., Holsinger, 2013; Kyriacou et al., 2020; Mancuso et al., 2020). It is also noted that the above-mentioned studies have mostly examined the receptive processing of FSs. By comparison, the processing of FSs in production tasks remains largely underexplored, particularly in second language (L2) contexts (Siyanova-Chanturia and Martinez, 2015; Siyanova-Chanturia and Lin, 2017). This is regrettable, since research into how L2 learners produce FSs can have important implications for theories concerning the production of L2 FSs as well as the teaching and learning of L2 FSs. The current study therefore aimed to investigate the (conscious) productive processing of FSs by L2 learners in writing.

In addition, there is a need to study FSs from L2 learners’ perspective. To date, studies investigating formulaicity in L2 have mostly defined FSs according to native-speaker norms such as authoritative dictionaries and corpus-derived measures, and examined how these idiomatic expressions are used and processed by L2 learners (Myles and Cordier, 2017). Nevertheless, there exists a potential paradox: the targeted FSs might not be known or familiar to L2 learners, thus not serving as holistic, formulaic units for them at all (Schmitt et al., 2004). This issue can be clarified by the important distinction between speaker-external and speaker-internal approaches to formulaicity (Wray, 2008). Speaker-external approaches study conventionalized expressions in the language outside the speaker, identified by external criteria such as formal properties and corpus frequency. Contrastively, speaker-internal or psychological approaches focus on sequences considered formulaic because they are psycholinguistic units for a particular speaker. Underscoring the speaker-internal approaches, this study distinguishes between internal and purely external FSs as the primary criterion of categorizing learners’ processing behaviors.

## 2 Literature review

This section reviews the definitions of FSs and their subtypes, the “holistic or compositional” debate on FS processing, and previous studies on the FS processing types in the learners’ production tasks.

### 2.1 Defining FSs

The most often-cited psycholinguistic definition of FSs was proposed by Wray (2002):

“a sequence, continuous or discontinuous, of words or other elements, which is, or appears to be, prefabricated: that is, stored and retrieved whole from memory at the time of use, rather than being subject to generation or analysis by the language grammar” (p. 9).

This definition characterizes the holistic property of FSs. However, as Cordier (2013) noted, it seems to suffer a self-contradiction: if the sequence is a discontinuous, flexible formulaic frame with slots for insertion, “it is difficult to conceive that no grammatical processing is taking place at all” (p. 20).

Concerning the identification of psychological FSs, Wray (2008) proposed 11 diagnostic criteria, including previous encounter with the precise formulation, which concerns the speaker’s acquisition experience of the FS. Moreover, Myles and Cordier (2017) maintained that a psychological FS should have a holistic quality: “semantic/functional unity or holistic mode of acquisition” (p. 20). The latter means that sequences can receive holistic status, if they are learned as wholes by learners. As can be seen, both Wray (2008) and Myles and Cordier (2017) considered learner’s previous acquisition experience of the FS as an important identification criterion.

Importantly, following Wray (2008) and Myles and Cordier (2017) called for a clear awareness of the difference between learner-internal and learner-external FSs. They posited that although there is considerable overlap between what is formulaic for a particular speaker and what is formulaic in the language around this speaker, these two constructs represent different phenomena, and should be investigated as such.

This study follows Wray’s (2002) convention to use FS as a coverall for not only psycholinguistic units but also sequences considered formulaic according to external criteria. To capture also external FSs, this study adopts her definition of FSs with modifications: a continuous or discontinuous sequence of words, which appears to be prefabricated, because it is a psycholinguistic unit for a particular learner and/or because it is a conventionalized expression in the language.

Furthermore, separate definitions have been proposed for internal FSs and external FSs. Following Wray (2008) and Myles and Cordier (2017), this study attaches importance to the learner’s previous acquisition experience in defining internal FSs. Besides, this study also deems it necessary to establish the holistic status of internal FSs according to the learner’s own psychological perception, since formulaicity is viewed as “fundamentally a psychological concept” (Hoey, 2005, p. 7). Therefore, a learner-internal FS is defined as: a continuous or discontinuous sequence of words, acquired previously and perceived as a whole by the learner, rather than being generated word-by-word at the time of use. In this definition, previous acquisition experience of FSs covers not only encountering or learning the FSs from previous linguistic input, but also the fusion of FSs by learners themselves. This is because fusion, which means that previously self-created strings become stored holistically through repeated use, was proposed as an important way of FS acquisition for L2 learners (Wray, 2002). This definition of learner-internal FSs intends to be exploratory and inclusive. It only claims some degree of holistic representation, which corresponds to some degree of entrenchment (Langacker, 1987; Schmid, 2007; Divjak and Caldwell-Harris, 2015), referring to “the process through which a structure becomes automated into a unit” (Wolter and Gyllstad, 2013, p. 452).

On the other hand, drawing on previous definitions in learner-external approaches of formulaicity (e.g., Qi and Ding, 2011; Jeong and Jiang, 2019; Yu, 2022), this study defines a learner-external FS as: a continuous or discontinuous sequence of words, which has a syntactically and semantically well-formed structure, and can be a conventional way of expressing something.

**Figure 1:**
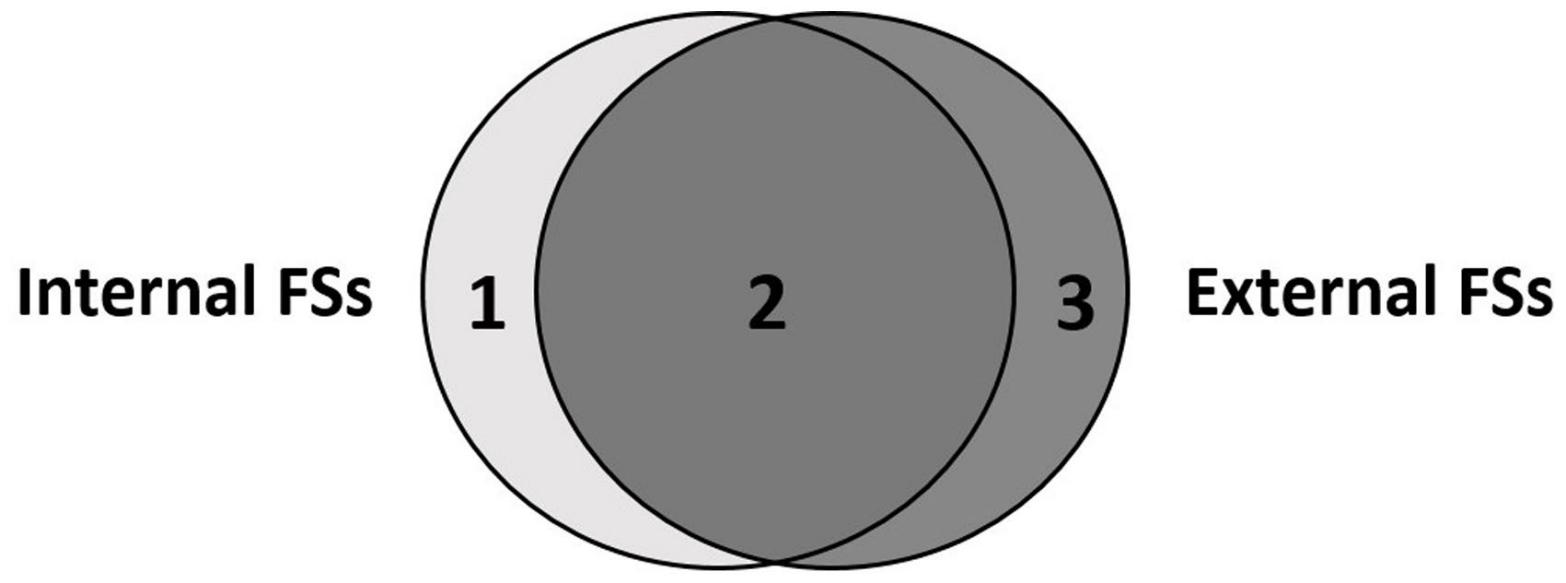
The difference between learner-internal and learner-external FSs.

Additionally, in light of the difference between learner-internal and learner-external FSs (see Figure 1), as emphasized by Myles and Cordier (2017), this study distinguishes between internal and purely external FSs as the primary criterion of categorizing learners’ processing behaviors.

As Figure 1 illustrates, although learner-internal and learner-external FSs may overlap considerably (area 2), there would be purely internal FSs (area 1) and purely external FSs (area 3). Purely internal FSs can be seen as idiosyncratic FSs, which are either self-fused strings or low frequency phrases memorized by their users. Such FSs are likely to be neglected by external approaches of formulaicity. Purely external FSs are those conventional FSs that are not perceived as wholes by learners either because of their high compositionality or their low or zero occurrence in the learners’ previous linguistic input. These FSs are isolated and termed as assembled FSs in this study, as they are perceived as being assembled word-by-word by the learners. This study does not distinguish between purely internal FSs (area 1) and overlap FSs (area 2) within internal FSs, since they are perceived as wholes indiscriminately from the learners’ perspective.

### 2.2 The “holistic or compositional” debate on FS processing

In psycholinguistic studies, there has been much debate on whether FSs are processed holistically or in a compositional manner. The holistic accounts see FSs as “long words” that are stored and processed holistically, assuming that the components of FSs are not analyzed and there would be no grammatical analysis during their use (e.g., Bobrow and Bell, 1973; Swinney and Cutler, 1979; Gibbs, 1980; Jackendoff, 2002). This assumption of holistic processing has been typically supported by empirical evidence of greater ease in processing FSs than matched non-formulaic phrases, such as shorter reaction time in grammaticality judgment tasks (e.g., Jiang and Nekrasova, 2007), and faster silent reading and articulation (e.g., Tremblay et al., 2011; Kim and Kim, 2012; Hallin and Van Lancker Sidtis, 2017). It has been claimed that FSs enjoy processing advantage because they can bypass the time-consuming syntactic analysis (Swinney and Cutler, 1979). However, this claim has come under criticism. Some researchers pointed out that the processing advantage of FSs did not indicate holistic storage, since it did not concern the relation between the parts and the whole (Arnon and Snider, 2010; Edmonds, 2014; Siyanova-Chanturia, 2015).

Contrastively, the compositional and hybrid accounts emphasize the compositional nature of FSs. Specifically, evidence shows that the literal meanings of component words can be activated during idiom processing (e.g., Cacciari and Tabossi, 1988; Glucksberg, 1993; Sprenger et al., 2006; Cacciari and Corradini, 2015; Beck and Weber, 2016; van Ginkel and Dijkstra, 2019; Kessler et al., 2020); idioms undergo the same regular syntactic analysis as nonidioms (e.g., Cutting and Bock, 1997; Holsinger, 2013); and frequency information of component words still affects the processing of even highly frequent collocations (Arnon and Cohen Priva, 2014; Wolter and Yamashita, 2018; Öksüz et al., 2020).

Importantly, the hybrid accounts view FSs as being holistic and compositional at the same time: while idioms are represented as wholes on some level of processing, they have their internal structures and can be syntactically analyzed on some other level. The holistic nature is reflected in their conventionality and the observation that they are processed faster and more accurately than non-formulaic controls. The compositional nature is revealed by the fact that some of them are decomposable and transparent. As Cieślicka (2010) put it, the apparent inconsistency in FS processing studies can be best resolved by the hybrid accounts of FS representation.

### 2.3 The hybrid models of idiom representation

**Figure 2:**
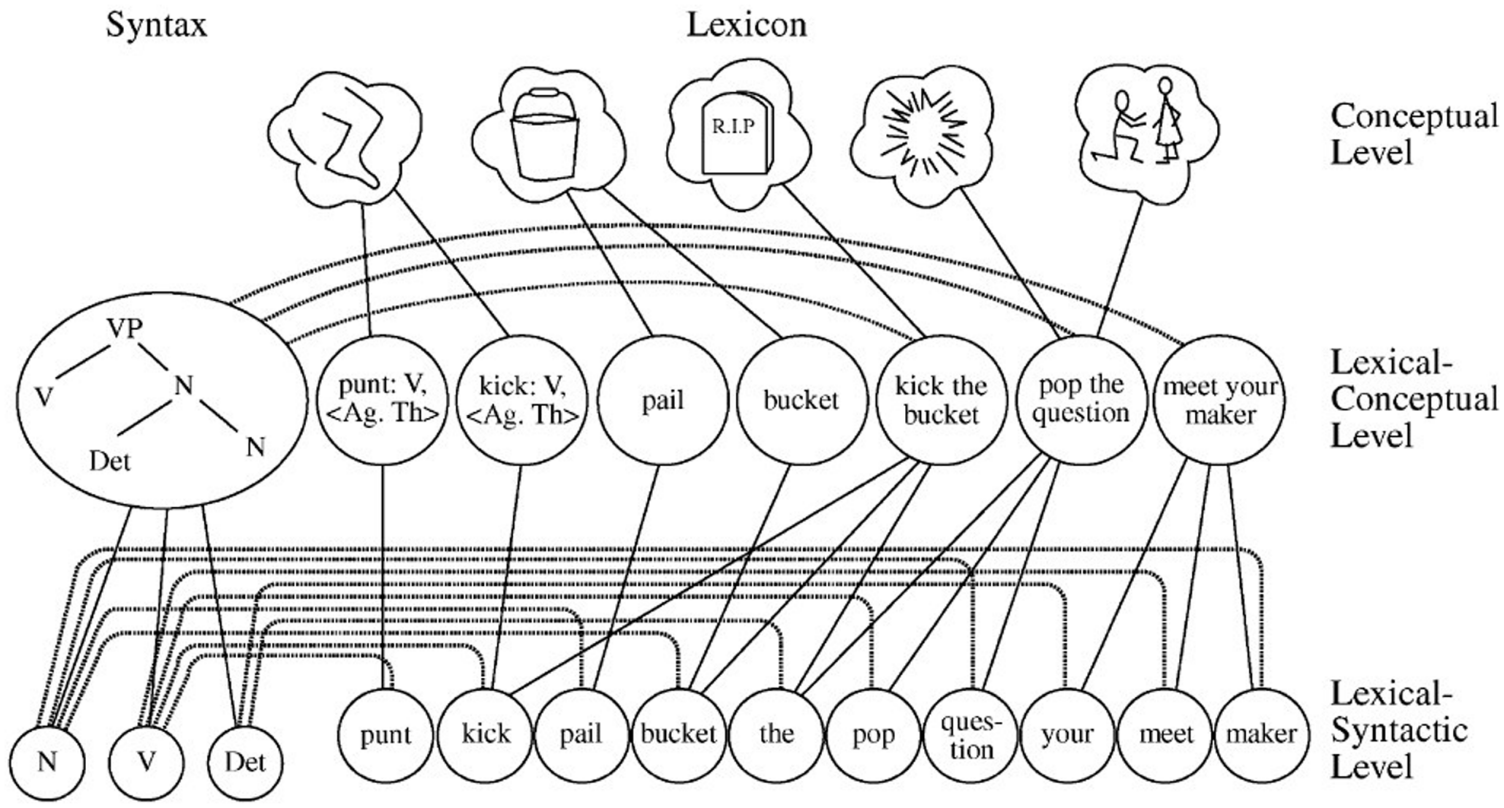
Model of the lexicon in Cutting and Bock (1997).

One influential model in the hybrid accounts is Model of the lexicon proposed by Cutting and Bock (1997), as shown in Figure 2. From top to bottom, the model consists of three processing levels: conceptual, lexical-conceptual and lexical-syntactic. Idioms are represented as holistic units on the lexical-conceptual level, each having its own lexical-concept node. Meanwhile, idioms are also composed of single words. Horizontally, the model distinguishes between lexicon and syntax. When the lexical-concept node of an idiom is activated, the activation spreads in two directions: one towards the single lemmas that constitute the idiom (lexicon-oriented); the other towards the syntactic information in the form of phrasal frames (syntax-oriented). As the model embraces the dualistic nature of idioms, it has been labeled as a hybrid model of idiom representation.

**Figure 3:**
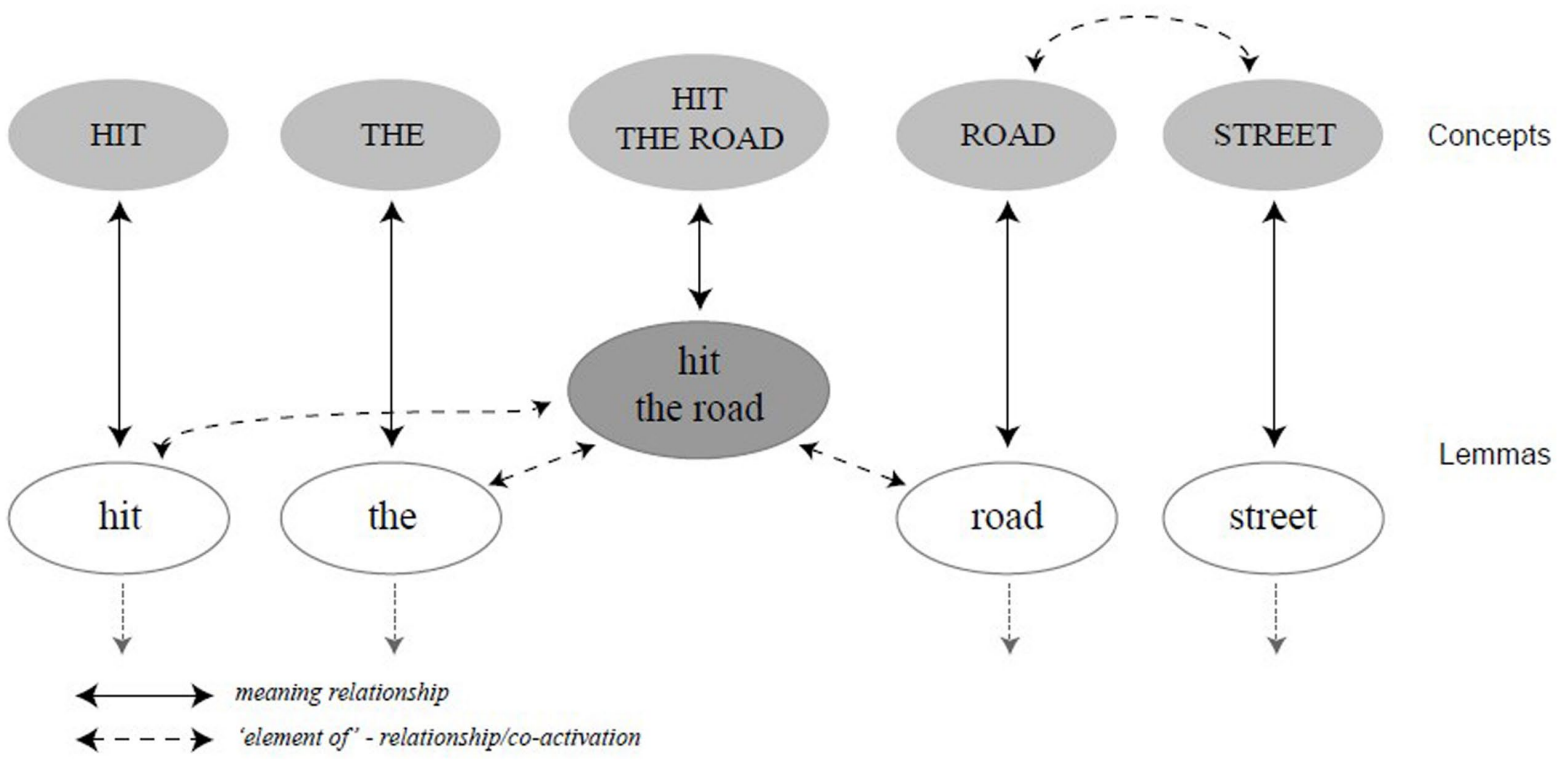
The superlemma model in Sprenger et al. (2006).

Later, Sprenger et al. (2006) proposed a modification of the hybrid model, as shown in Figure 3. Specifically, they introduced a *superlemma*, defined as a representation of the idiom on the lemma level, which is a sublevel of the lexical-syntactic level, *lemma* referring to representation of a word’s semantic and syntactic information plus a pointer to the word form (see Levelt, 1989; Roelofs, 1992; Jiang, 2000). The adapted model has been termed as the superlemma model, in which idiom production follows the same rules of competition and selection as single words do. For example, the superlemma *hit the road* might compete with *leave*, since there might be competition among co-activated lemmas for the same concept. Importantly, Sprenger et al. (2006) contended that the hybrid view can be seen as a general production model of FSs, which may vary in degrees of fixedness, transparency and compositionality.

The hybrid view offers a good solution to the “holistic or compositional” debate on FS processing. Nevertheless, there is still room for improvement. First, the hybrid models are proposed for the production of idioms in the first language (L1). Hence, how they can be adapted for L2 contexts remains to be explored. Second, the basic processing levels in the hybrid models need further specification. The inquiries include such as: What are the chances of co-retrieval of an FS and other lexical items? What if the FS could not be retrieved in its entirety due to inadequate entrenchment in the mental lexicon? How many FSs would be used with syntactic modification? What is the proportion of (conscious) holistic processing on the lexical-syntactic level? Taking these concerns into consideration, the current study examines how FSs are processed by L2 learners in writing. In this way, it could add to the specifications of the lexical-syntactic level in previous hybrid models for L2 contexts.

### 2.4 FS processing types in the learners’ production tasks

So far, only a few studies have investigated the productive processing of FSs. Three of them identified different FS processing types in the learners’ production tasks. The earliest one (Spöttl and McCarthy, 2004) explored how multilingual learners processed FSs in a translation task across three or four languages (from L2 into the L1 and then into the third language and/or the fourth language, which can be seen as L2s in a broad sense). Based on think-aloud protocols, three processing types were identified: automatic processing (fluent translation without repetition or evaluation); synthetic evaluative processing (L2 FS repeated and various responses produced and evaluated after a failed attempt at translation); and analytic evaluative processing (FS component words repeated to start the search after a failed attempt at translation). The first two types were described as holistic processing. It was found that synthetic evaluative processing was the most frequent; and automatic processing was employed only occasionally.

In Xu (2010), the participants were English majors from two proficiency levels, writing about the most unforgettable experience in their life. Based on think-aloud protocols, three FS retrieval types were proposed: automatic retrieval (smooth flow of thought), “tip-of-the-tongue” (failed attempt to retrieve the complete form), and piecemeal construction (step-by-step retrieval). Automatic retrieval was found to be the dominant type of retrieval, occupying 81 and 94% for the two groups. Furthermore, higher-proficiency learners had significantly more automatic retrieval and less piecemeal construction than their lower-proficiency counterparts.

Using computer keystroke recordings, Yuan and Xu (2016) investigated the production of FSs by university students in their argumentative L2 writing, and identified three processing types: automatic processing (fluent and fast-rate production, described as holistic processing), semi-automatic processing (fluent but slow-rate production), and controlled processing (dis-fluent production). The authors noted that automatic processing only accounted for 41.98%, while controlled processing was the most frequent. This result differed from Xu’s (2010) finding that automatic retrieval was the most frequent, a possible reason being that, compared with the participants in Yuan and Xu (2016), those in Xu (2010) probably wrote on a more familiar topic, thus retrieving more FSs automatically. Topic familiarity has been shown to affect various aspects of learners’ production performance, such as fluency (Bui, 2014), lexical complexity (Yang and Kim, 2020; Bui, 2021) and density of FS use (Cordier, 2013). Drawing on Levelt’s (1989) model, Bui (2014) suggested that topic familiarity may affect processing at both the Conceptualization and the Formulation stages, resulting in faster access to familiar information and faster retrieval of memorized chunks, which are crucial for the production of FSs.

To summarize, previous studies have proposed tripartite categorizations of FS processing types in learners’ production tasks, thus shedding light on the productive processing of L2 FSs. Nevertheless, there are still unresolved issues. First, the processing types involved in L2 FS production await further investigation, as previous studies have yielded inconsistent findings with respect to the proportion of automatic processing or retrieval in L2 FS production, and none of them classified systematically the processing types into holistic or compositional processing. Furthermore, in those studies, the FSs in the learners’ language production were identified on the basis of the researchers’ judgments, so they might not necessarily match the holistic units from the learners’ perspective. Besides, those studies relied on think-aloud method or computer recordings, which may have problems: performing think-aloud might interfere with the normal thinking process (Stratman and Hamp-Lyons, 1994; Sasaki, 2000); the computer-recorded typing might not necessarily mirror the participants’ mental activity.

Second, it would be worth exploring the influence of learners’ L2 proficiency on their productive processing of FSs. Xu (2010) has suggested the positive relationship between the degree of automaticity in FS production and proficiency development. Besides, the inconsistency in previous findings might stem from differences in the language proficiency of the participants.

Third, another issue concerns the influence of topic familiarity on learners’ productive processing of FSs, as is indicated by the difference between the findings in Yuan and Xu (2016) and Xu (2010).

To address the foregoing unresolved issues, this study investigated L2 learners’ productive processing of FSs from their own perspective, using the method of video stimulated recall (VSR). While doing so, it also took into consideration the possible effects of L2 proficiency and topic familiarity. The research questions of this study include:

1. How do L2 learners process FSs in writing?
1a. What are the major FS processing types and their frequency/proportion?
1b. Which FS processing types can be seen as holistic processing or compositional processing?
2. What are the effects of L2 proficiency on the learners’ productive processing of FSs?
3. What are the effects of topic familiarity on the learners’ productive processing of FSs?

Based on the review of previous processing models and empirical studies, the following hypotheses were proposed.

*Hypothesis 1*: The learners’ FS processing types could be categorized on the lexical and syntactic levels (Cutting and Bock, 1997; Sprenger et al., 2006; Xu and Ding, 2010). There would be more holistic processing than compositional processing on each level, as the production of FSs appeared automatic and effortless in most cases according to the learners’ verbal reports (Xu, 2010).

*Hypothesis 2*: Higher L2 proficiency would lead to more holistic processing on each level, as L2 proficiency is positively associated with the degree of automaticity in FS production (Xu, 2010).

*Hypothesis 3*: Higher topic familiarity would lead to more holistic processing on each level, as topic familiarity is associated with better performance in learners’ production tasks (He and Shi, 2012; Bui, 2014).

## 3 Methodology

### 3.1 Design

The current study was primarily qualitative, supplemented by quantitative analysis. It involved collecting and analyzing qualitative data in the first phase of the study and analyzing quantitative data in the second phase. Qualitative analysis was used to delineate learners’ processing types while quantitative analysis was conducted to detect if there was any significant difference between the two proficiency groups, or between the two writing tasks. Table 1 provides an overview of the research design. A detailed account follows thereafter.

**Table 1:** Overview of the research design.

Participants	10 freshmen of two L2 proficiency levels
Instruments	Two writing tasks differing in topic familiarity; Training material for students’ FS identification; Video stimulated recall (VSR)
Data collection	Session 1: the student writing on the familiar topic→ the training of FS identification→ the student and the researcher identifying FSs separately→ the student and the researcher comparing the two versions to locate potential internal FSs and assembled FSs → the training of VSR → the student performing VSR
	Session 2: the student writing on the unfamiliar topic (The same procedure was repeated except the trainings)
	Session 3: the interview about FS acquisition experience
Data preparation	Transcribing verbal recordings→ ascertaining internal FSs and assembled FSs
Data coding and analysis	Coding of FS processing types (Qualitative analysis) → tallying the descriptive statistics→ non-parametric tests to examine the effects of proficiency and topic familiarity (Quantitative analysis)

### 3.2 Participants

The participants in this study were ten first-year undergraduate students (L1 Chinese, 8 males and 2 females, aged between 18 and 19) at their second semester from a university in East China. At the beginning of their first semester, they all took a comprehensive placement test to be enrolled in a three-level English program for non-English majors (Level 1 presenting the highest proficiency in this population). The English courses for Level-1 students were *College English Reading and Writing* (Level-1) and *College English Listening and Speaking* (Level-1), each having 2 hours of instruction per week. Similarly, Level-3 students attended these two types of courses for Level 3, with the same hours of instruction. Five participants were from Level 1 (hereafter HS1 to HS5 for the five higher-proficiency students), and the other five from Level 3 (hereafter LS1 to LS5 for the five lower-proficiency students). Just prior to the experiment, they all took the College English Test-Band 4, which is a nationwide standardized proficiency test in China for college students. The five higher-proficiency students received an average score of 637.8 (SD = 23.7) out of 710, while the five lower-proficiency students received an average score of 554.8 (SD = 43.1). Therefore, they can be deemed as advanced level and upper intermediate level respectively, as students scoring above 530 were considered as upper-intermediate and advanced (Kessler et al., 2021). Their teachers judged the students’ proficiency to approximate level C1 (lower-advanced) and level B2 (upper-intermediate) of the Common European Framework of Reference (CEFR), respectively.

In addition, all of the participants had been raised in China and none had the experience of living abroad. They started learning English from the first or the third grade at primary school (average starting age: 7 (SD = 1.22) for higher-proficiency students and 7.4 (SD = 1.52) for lower-proficiency students; average years of formal instruction: 12.2 (SD = 1.3) for higher-proficiency students and 11.8 (SD = 1.3) for lower-proficiency students). These participants were recruited through random invitation with the help of their teachers and received stationery as gifts for their participation.

### 3.3 Instruments

#### 3.3.1 Writing tasks

Two argumentative writing tasks differing in topic familiarity were used in this study. The argumentative essay was chosen because it is probably the most common genre practiced at the undergraduate level (e.g., Jiang, 2015; Chen, 2019; Shin, 2019). First, 11 topics were selected from a large number of writing tasks in English tests commonly taken by college students, such as Test of English as a Foreign Language (TOEFL) and The International English Language Testing System (IELTS). Then, 16 freshmen completed a questionnaire to evaluate the familiarity of each topic on a 7-point Likert scale. Half of them were Level-1 students from a parallel class as the HS participants, while the other half were Level-3 students from a parallel class as the LS participants. Thus, they can be seen as representative of the participants in this study. According to the survey results, two topics were chosen (familiarity scores: 5.75 versus 3.38). In both tasks, the participants were required to write about 150–200 words within 35 min. The writing prompts are as follows (written in Chinese in the experiment to avoid any text borrowing):

1. In this fast-paced age, people often confront various kinds of pressure, and college students are no exception. Please write a short essay of 150 to 200 words discussing the reasons for college students’ pressure and the solution to it.
2. With the development of economic globalization, the global competition has become more and more fierce. Some people suggest that, to protect the national economy, we should encourage the purchase of domestic products, and limit the purchase of foreign products. How do you view this suggestion? Please write a short essay of 150 to 200 words discussing your viewpoint and giving your reasons.

#### 3.3.2 Training material for students’ FS identification

The current study relied on students’ judgments in the identification of learner-internal FSs. After the first writing task, the training of FS identification ensued. A three-part PowerPoint presentation was given to the students in Chinese (see the presentation in Supplementary material). The first two parts present a definition and a categorization of FSs in easy-to-understand language, supplemented by specific examples. The third part presents the identification criteria: at least two words in length; previously learned, encountered or used (including previously self-created expressions which were frequently used later); and being perceived as a whole (having impression of the holistic form, rather than assembling the expression word-by-word on the spot). Students’ identification of FSs can serve as a useful way to explore what is formulaic in their mind, since it has been found that laypeople’s intuitive judgments of formulaicity are valid (Wulff, 2008; Lin, 2018), and L2 learners’ intuitions are a reliable predictor of their idiom knowledge (Hubers et al., 2020).

#### 3.3.3 Video stimulated recall (VSR)

VSR interview is a technique for investigating the participants’ cognitive processes by promoting them to recall their thinking while playing video-recordings of their own behaviors (Dempsey, 2010). This technique has been proved highly effective in process-oriented writing studies (e.g., Abdi Tabari, 2022). It is suggested that VSR be conducted as soon as practicable to prevent recall failure (Dempsey, 2010; Gass and Mackey, 2017). Upon the completion of the writing and the FS identification (as will be illustrated below), the participants were provided with instructions and demonstrations on how to perform VSR. Then they performed VSR while watching the video-recordings of their writing process captured by the screen recording software Camtasia Studio.^1^ During the recall, the video could be paused or played back to allow for detailed explications. The participants were prompted by questions such as “What were you thinking at that moment?” “Why did you make this change?” “Why did you pause here?” and so on. Besides, after the recall of each paragraph, the student also recalled the production process of each FS according to the researcher’s prompt questions (see the prompt questions in Supplementary material).

### 3.4 Data collection

Data collection was carried out in three sessions for each participant on a one-to-one basis. In the first session, the student wrote about the familiar topic in a Word file (without auxiliary functions) on a computer. Meanwhile, the software Camtasia Studio was used to record the writing process on the screen. The next was the training of FS identification. After that, the student and the researcher identified the FSs, individually on separate computers. The student was required to mark in red the expressions they considered as FSs, while the researcher identified potential external FSs according to the criterion of structural completeness and semantic unity. Then, collectively, comparison was made between the two annotated versions. Sequences identified by the researcher but not the student were rechecked immediately, and then marked in different colors by the student: those acknowledged as FSs by the student were marked red since they had been missed simply because of overlooking, while those that the student claimed as being assembled from scratch were marked blue. Consequently, the “red” sequences were potential internal FSs to the student, while the “blue” sequences (researcher-only sequences) were potential assembled (purely external) FSs to him or her. Then, the student performed VSR for the FS production processes.

Three days later, in the second session, the writing topic changed to an unfamiliar one. The same procedure was repeated, except the training of FS identification and VSR. One day or several days thereafter, in the third session, the students were interviewed about their acquisition experience of the FSs they had identified. The interview began with some questions concerning the student’s FS acquisition experience in general, and then the acquisition experience of each FS was inquired with a series of questions (see the interview guide in Supplementary material).

### 3.5 Data preparation

All the verbal recordings were transcribed by the first author with the help of the software Iflyrec.^2^ As a next step, the potential internal FSs and assembled FSs needed to be ascertained for their formulaic status.

#### 3.5.1 Ascertaining internal FSs

The interview data about FS acquisition experience were used to ascertain the formulaic status of internal FSs, that is, to check if the FSs identified by the students had been acquired previously as wholes by them. Based on the interview data, this study classified the students’ FS acquisition experience into four categories: 1. deliberate memorization; 2. incidental learning without a particular intention to learn; 3. brief noticing or semi-intentional learning; and 4. fusion (acquiring the FS through self-construction and later frequent use).^3^ The formulaic status of an internal FS was ascertained if the student’s acquisition experience of it belonged to one of the four categories. Note that the internal FSs might be erroneous (e.g., *handle with), as such erroneous forms probably had been entrenched in the students’ mental lexicon by repeated use.

#### 3.5.2 Ascertaining assembled FSs

In identifying external FSs, we followed the FS identification method in Qi and Ding (2011) with some modifications. The criteria are as follows: 1. being composed of two or more than two words and having structural completeness and semantic unity; 2. being contained in the *Longman Dictionary of Contemporary English*^4^ or *Oxford Collocation Dictionary of English*^5^; or being listed as a collocation or cluster in the Corpus of Contemporary American English; 3. being confirmed by native speaker intuition.

During the FS identification immediately after each writing, the first criterion was applied by the researcher to locate external FSs. To ascertain the formulaic status of potential assembled FSs, the second and the third criteria needed to be applied to determine whether they qualified as authentic FSs in the language. Note that if the sequence resembled the standard form considerably, it was treated as an inappropriately assembled FS (e.g., *life quality/*quality of life*). The first author and a research assistant checked separately the potential assembled FSs in the dictionaries and the corpus. The differences in the results were settled through discussion and a unanimous list of FSs was reached. Finally, the FS list was presented to a native speaker for further confirmation. It turned out that all the FSs on the list were confirmed by native speaker intuition, as they had been carefully checked against authoritative sources including the dictionaries and the corpus.

### 3.6 Data coding and analysis

Based on the students’ VSRs and computer recordings, iterative analysis was carried out to categorize the students’ processing behaviors during their FS production. In line with previous models of language representation and production (Cutting and Bock, 1997; Sprenger et al., 2006; Xu and Ding, 2010), two processing levels were distinguished hierarchically: lexical and syntactic. These two levels can be seen as sub-levels of the lexical-syntactic level in Cutting and Bock (1997).

At the lexical level, the FS basic form is retrieved or assembled by the student. It is similar to the lexical retrieval stage in Xu and Ding (2010), where writers make efforts to retrieve the lexical items needed to convey the intended meaning. In the current study, categorization on this level primarily draws upon the distinction between internal FSs and assembled FSs, with the former being retrieved on the basis of the student’s impression of the holistic phrasal forms, while the latter being constructed word-by-word without a holistic base and coinciding with a conventionalized expression in the language. Then, within internal FSs, further categorization is made according to the literature (Xu, 2010) and the data of the present research. It is noteworthy that “the basic form” is conceptualized differently for internal and assembled FSs. For the former, it refers to the holistic form of the FS in the student’s mind, that is, the form that the student retrieves as a whole in the first place. These basic forms are delineated according to the students’ description of the phrasal form that appeared in their mind first for a certain meaning. For assembled FSs, the basic form refers to the student’s combination of individual words that coincides with an authoritative expression in the dictionaries and the corpus, such as *feel nervous*. In a sense, the FS basic form resembles superlemma in Sprenger et al. (2006). Nevertheless, it is the basic form of a holistically acquired phrase or a word combination from the student’s perspective, rather than in linguistic terms. Actually, the FS basic forms in this study might not be linguistically lemmatized forms as would appear at the beginning of a dictionary entry. For example, *ranging from…to* and *are easily to* were retrieved as FS basic forms.

At the syntactic level, the FS basic form is embedded in the text either intact (intact integration) or with modification (syntactic modification) by the student. It is similar to the formal integration stage (Xu and Ding, 2010), where writers attend to the formal features of lexical items and embed them in specific contexts. In the current study, intact integration is identified if the FS basic form remains the same in the written product; syntactic modification is detected according to the student’s description of the modification they made to the FS basic form in their conscious mind. For internal FSs, the unit of modification is the entire FS, and the modification can be made by lexical and morphological means (e.g., *be different from*→*is greatly different from*). For assembled FSs, the unit of modification is the individual component words, so the modification can only happen by morphological means (e.g., *feel nervous*→*feels nervous*).

After the coding scheme had been developed, the first author and a research assistant coded separately four randomly selected VSRs, and the inter-coder reliability was 0.92. The differences were resolved through discussion. Then, the first researcher coded the remaining data.

After the coding was done, the frequency and percentage of each FS processing type in each composition were tallied. Considering the small sample size, the current study conducted non-parametric tests using SPSS 25 for the quantitative analysis. The two independent variables include one between-participant variable (two proficiency levels) and one within-participant variable (familiar and unfamiliar topics). The dependent variable—learners’ FS processing—was measures in terms of frequency and percentage of FS processing types. Specifically, based on the descriptive data, Mann–Whitney U tests (Two-independent samples tests) were run to detect variances between the two proficiency groups, and Wilcoxon Signed Ranks tests (Two-related samples tests) to detect variances between the two writing tasks.

## 4 Results

### 4.1 Overall description of L2 learners’ processing of FSs in writing

#### 4.1.1 Major FS processing types and their frequency/proportion

The learners’ FS processing types are categorized on two processing levels: lexical and syntactic. On the lexical level, drawing on Xu’s (2010) taxonomy of FS retrieval types, the current study identified five FS processing types, i.e., single retrieval, parallel retrieval, part-to-whole retrieval, “din in the head” and online assembly. The first four types describe the processing of internal FSs, while online assembly denotes the processing of assembled FSs.

Single retrieval means that the FS is retrieved fluently in its entirety as the single choice for a certain meaning. For example:

Upon seeing the writing topic, I felt that it is about a common phenomenon. So I came up with a chunk “it is universally acknowledged that.” (HS5-Familiar)

Parallel retrieval means that the FS is retrieved simultaneously with other expression (s) for the same meaning. For example:

For 各种各样的压力 [all kinds of pressure], several expressions flashed up in my mind, like “various” and “a variety of.” I used “all kinds of” because it was more familiar to me. (HS1-Familiar)

Part-to-whole retrieval means that the FS is retrieved not in its entirety, but rather in a part-to-whole manner. Specifically, the writer retrieved a part of the FS first, and then retrieved the remaining part either fluently or laboriously. This indicated that the component words of FSs do not always have equal weighting, with some being more salient and more easily retrievable than others. In the following example, *take charge of* was retrieved by laborious part-to-whole retrieval, as the student had struggled to recall the final word of it:

college students need to take charge of themselves.

I was hesitating between “for” and “of” for quite a while. (HS4-Familiar)

“Din in the head” means that the target FS cannot be successfully retrieved at the moment despite the student’s retrieving effort. To be specific, the students had an ideal FS in their mind for the current use, but they were unable to retrieve its form successfully. The term “din in the head” was originally defined as “the sense of having the language available for use” (Krashen, 1985, cited from Cohen, 1998, p. 244). This term, rather than “tip of the tongue” (Xu, 2010), is used in the current study, as it implies only a weak memory trace of the expression. Indeed, students may come across the disappointing situation that the memory trace of the desired FS was too weak that they failed to retrieve its form. “Din in the head” differs from part-to-whole retrieval in that it denotes failed retrieval, though they both entail construction efforts. For example:

I wanted to express 分轻重缓急 [get your priorities right] and thought of a newly learned chunk for this meaning, but I couldn’t recall it, so I gave up. (HS2-Familiar)

Online assembly means that the FS is assembled word-by-word on the spot. These FSs are the researcher-only FSs, i.e., only identified by the researcher and deemed as being assembled or improvised by the students. Online assembly can also be fluent or laborious, depending on whether the student had difficulties during the assembling process. Notably, online assembly differs from part-to-whole retrieval and “din in the head” in that it denotes word-by-word construction from scratch, while the other two denote construction on the basis of some vague or “worn-out” memory traces. Consider an example of fluent online assembly:

(*reduce pressure*) To express 减轻压力, I thought of 压力 [pressure] first, and then 减轻 [reduce]. Then I judged whether they could collocate. As I thought they could, I put them together. (LS4-Familiar)

Among the total 45.95 FSs retrieved or assembled during a writing task averagely, single retrieval was the most frequent (mean frequency/percentage = 27.05/58.16%), followed by online assembly (10.20/23.32%), parallel retrieval (5.55/11.44%), part-to-whole retrieval (2.70/6.09%) and “din in the head” (0.45/0.99%). Furthermore, the accumulated frequency/percentage of internal FSs (all the categories except online assembly) is 35.75/76.68%.

On the syntactic level, a distinction was made between intact integration and syntactic modification, depending on whether the FS basic forms were used intact or with modification. Intact integration means that the FS basic form is embedded intact in the text without any modification. For instance:

“A good choice” came out directly, and I made no change to it. Ah, why didn’t I think over “good”? “Good” can certainly be changed for a better word. What a pity. (LS4-Familiar)

Interestingly, it was found that the FS basic forms are not necessarily linguistically lemmatized forms. Rather, they might contain inflected words. For example:

I always use “we are supposed to.” Lots of writings are about suggestions. Although the collocation is “be supposed to,” I commonly use “are supposed to” directly, and seldom use “be supposed to.” (LS2-Familiar)

As illustrated above, the FS *we are supposed to*, fully specified in grammatical features, was embedded intact in the text. This suggests that such grammatical markers may have been frequently used with the particular FS to the extent that they have become an integral part of it. Either the inflected forms have also been stored in the mental lexicon, or the syntactic-morphological operations have become so automatized that they do not need any conscious effort. This is consistent with the hypothesis that FSs may be stored at different levels of abstraction (Ambridge and Lieven, 2011; Cordier, 2013; Wulff, 2018). For example, Cordier (2013) found that while learners seem to store abstract formulaic frames, they may also have automatized some fixed, specific sequences.

On the other hand, syntactic modification means that the FS basic form is modified in one way or another according to the specific context. It was found that learners’ syntactic modification could happen in the morphological aspect such as person, tense, participle, and determiner, or in the lexical aspect including addition, substitution and omission of words within the FS, or in both. For example:

Morphological modification (participle):

a “native” product may *has its raw materials originating from other* countries.

I thought of “originate from.” I knew participle should be used, yet hesitating between present participle and past participle. (HS3-Unfamiliar)

Lexical modification (substitution):

*I’m appreciate to share some opinions about it with you.

This is a frequently used sentence pattern. I thought “glad” was quite clichéd, so I substituted it with “appreciate.” (LS1-Unfamiliar)

Among the 41.45 FSs embedded per text on average, intact integration (mean frequency/percentage = 27.25/66.53%) was much more frequent than syntactic modification (14.20/33.47%). Note that the frequency of embedded FSs was lower than that of retrieved/assembled FSs (45.95). This is because some FSs, albeit being retrieved or assembled on the lexical level, were obsoleted without being embedded, thus failing to reach the syntactic level. Furthermore, internal FSs with intact integration were the most frequent (mean frequency/percentage = 19.55/46.79%), followed by internal FSs with syntactic modification (11.75/27.78%), assembled FSs with intact integration (7.70/19.7%), and finally assembled FSs with syntactic modification (2.45/5.69%).

#### 4.1.2 Dividing the FS processing types into holistic or compositional processing

In this study, holistic processing is conceptualized as the processing that does not involve writers’ conscious, overt syntactic analysis of the FSs into component words. In the two-layered categorization, on the lexical level, single retrieval and parallel retrieval can be seen as holistic processing in a sense. By contrast, the other types should be regarded as compositional processing, since they all entail some constructional efforts. On the syntactic level, intact integration of internal FSs can be seen as holistic processing in a sense (though there might be some minimal syntactic analysis), while syntactic modification of internal FSs should be regarded as compositional processing, since it is the overt manifestation of syntactic analysis. Besides, since assembled FSs were not perceived as holistic units by the learners, all the integration of assembled FSs belongs to compositional processing. Note that the difference between holistic processing and compositional processing is a matter of degree, as holistic processing is a gradable concept (Boers et al., 2006) and idiomaticity is a scalar property (Wulff, 2008).

Consequently, the results of the present study can give an indication of the relative proportion of holistic versus compositional processing on each level. On the lexical level, holistic processing may account for 69.60% (the accumulated percentage of single and parallel retrieval), which is essentially determined by the percentage of internal FSs (76.68%), since internal FSs retrieved through compositional processing (part-to-whole retrieval and “din in the head”) are quite scarce. On the syntactic level, holistic processing may account for 46.79% (the percentage of intact integration of internal FSs). Inversely, compositional processing may account for 30.40% on the lexical level and 53.21% on the syntactic level.

### 4.2 The productive processing of FSs by the two proficiency groups

To answer the second research question, comparisons were made between the two proficiency groups with respect to the productive processing of FSs. Table 2 presents the descriptive results and the statistical test results concerning the between-group comparison of the five FS processing types on the lexical level. Mann–Whitney U tests showed that higher-proficiency students had significantly more single retrieval (*Z* = −2.656, *p* < 0.01) and parallel retrieval (*Z* = −2.621, *p* < 0.01) in frequency, while lower-proficiency students had significantly more online assembly in percentage (*Z* = −1.978, *p* < 0.05). Furthermore, the accumulated frequency/percentage of internal FSs was 42.8/81.76% for higher-proficiency students and 28.7/71.59% for lower-proficiency students, while the frequency/percentage of assembled FSs was 9.4/18.24% for higher-proficiency students and 11.0/28.41% for lower-proficiency students. This indicates that compared with lower-proficiency students, the higher-proficiency group were more likely to retrieve prefabricated expressions from their mental lexicon, rather than assemble FSs from scratch.

**Table 2:** Between-group comparison on the lexical level.

Categories	Frequency				Percentage (%)			
	High	Low	Z	Sig.	High	Low	Z	Sig.
Single retrieval	31.6 (7.4)	22.5 (6.5)	−2.656	0.008**	60.02 (5.98)	56.30 (11.33)	−0.643	0.520
Parallel retrieval	7.9 (4.8)	3.2 (2.3)	−2.621	0.009**	14.91 (7.93)	7.97 (5.90)	−2.015	0.044*
Part-to-whole retrieval	2.7 (2.1)	2.7 (1.8)	−0.193	0.847	5.64 (5.40)	6.53 (3.95)	−0.874	0.382
Din in the head	0.6 (0.8)	0.3 (0.5)	−0.717	0.473	1.19 (1.64)	0.79 (1.29)	−0.534	0.593
Online assembly	9.4 (2.7)	11.0 (3.5)	−1.031	0.303	18.24 (4.72)	28.41 (10.74)	−1.978	0.048*

High, Higher-proficiency group; Low, Lower-proficiency group; Standard deviation is given in parentheses. *, significant at *p* < 0.05; **, significant at *p* < 0.01.

Table 3 reports the descriptive results and the statistical test results concerning the between-group comparison of the two FS processing types on the syntactic level. Mann–Whitney U tests revealed that the two groups had significant differences on this level: higher-proficiency students employed significantly less intact integration in percentage (*Z* = −2.960, *p* < 0.01), and made significantly more syntactic modifications in frequency (*Z* = −3.600, *p* < 0.001) and percentage (*Z* = −2.960, *p* < 0.01). Table 4 further demonstrates that these differences mainly lie in the processing of internal FSs: higher-proficiency students made significantly more modifications to internal FSs (Frequency, *Z* = −3.413, *p* < 0.01; Percentage, *Z* = −2.613, *p* < 0.01).

**Table 3:** Between-group comparison on the syntactic level.

Categories	Frequency				Percentage (%)			
	High	Low	Z	Sig.	High	Low	Z	Sig.
Intact integration	27.6 (5.7)	26.9 (6.0)	−0.152	0.879	59.85 (8.66)	73.21 (6.34)	−2.960	0.003**
syntactic modification	18.7 (5.7)	9.7 (2.3)	−3.600	0.000***	40.15 (8.66)	26.79 (6.34)	−2.960	0.003**

High, Higher-proficiency group; Low, Lower-proficiency group; Standard deviation is given in parentheses. **, significant at *p* < 0.01; ***, significant at *p* < 0.001.

**Table 4:** Between-group comparison within internal FSs and assembled FSs on the syntactic level.

Categories		Frequency				Percentage (%)			
		High	Low	Z	Sig.	High	Low	Z	Sig.
Internal FSs	Intact integration	21.1 (6.0)	18.0 (6.1)	−1.138	0.255	45.07 (7.10)	48.51 (9.35)	−1.099	0.272
	Syntactic modification	15.7 (4.0)	7.8 (3.3)	−3.413	0.001**	34.23 (8.45)	21.34 (8.90)	−2.613	0.009**
Assembled FSs	Intact integration	6.5 (3.0)	8.9 (2.6)	−1.638	0.101	14.79 (7.58)	24.70 (7.88)	−2.196	0.028*
	Syntactic modification	3.0 (2.7)	1.9 (1.2)	−0.695	0.487	5.92 (4.30)	5.45 (3.94)	−0.038	0.970

High, Higher-proficiency group; Low, Lower-proficiency group; Standard deviation is given in parentheses. *, significant at *p* < 0.05; **, significant at *p* < 0.01.

Furthermore, on the lexical level, the proportion of holistic processing was 74.93% for higher-proficiency students and 64.27% for lower-proficiency students. This difference resulted from higher-proficiency students’ retrieval of more internal FSs than lower-proficiency students. On the syntactic level, the proportion of holistic processing was 45.07% for higher-proficiency students and 48.51% for lower-proficiency students. Despite retrieving more internal FSs, higher-proficiency students nevertheless made more modifications to them, thus reducing holistic processing on the syntactic level. Taken together, the results show that higher L2 proficiency may lead to higher proportion of holistic processing on the lexical level but not the syntactic level.

### 4.3 The productive processing of FSs in the two writing tasks

To answer the third research question, comparisons were made between the two writing tasks with respect to the productive processing of FSs. Table 5 presents the descriptive results and the statistical test results concerning the between-task comparison of the five FS processing types on the lexical level. It can be seen that the two tasks resembled each other considerably on this level. Wilcoxon Signed Ranks tests showed that the only significant difference was the higher frequency of single retrieval in the familiar-topic writing (*Z* = −2.524, *p* < 0.05). This contributes to the higher frequency of internal FSs in the familiar-topic writing than in the unfamiliar-topic writing (39.5 versus 32). Nevertheless, the proportions of internal FSs in the two tasks were almost the same (77.25% versus 76.12%), since there were also more assembled FSs in the familiar-topic writing (10.8 versus 9.6).

**Table 5:** Between-task comparison on the lexical level.

Categories	Frequency				Percentage (%)			
	Familiar	Unfamiliar	Z	Sig.	Familiar	Unfamiliar	Z	Sig.
Single retrieval	30.7 (9.0)	23.4 (5.8)	−2.524	0.012*	60.40 (8.61)	55.92 (9.31)	−1.581	0.114
Parallel retrieval	5.7 (5.1)	5.4 (3.9)	−0.141	0.888	10.72 (8.14)	12.16 (7.56)	−0.833	0.405
Part-to-whole retrieval	2.5 (1.9)	2.9 (2.0)	−0.539	0.590	4.86 (3.55)	7.32 (5.40)	−1.053	0.292
Din in the head	0.6 (0.8)	0.3 (0.5)	−1.000	0.317	1.27 (1.70)	0.72 (1.17)	−0.850	0.395
Online assembly	10.8 (3.0)	9.6 (3.3)	−1.196	0.232	22.76 (9.42)	23.89 (10.28)	−0.416	0.677

Familiar, Familiar-topic writing; Unfamiliar, Unfamiliar-topic writing; Standard deviation is given in parentheses. *, significant at *p* < 0.05.

Table 6 reports the descriptive results and the statistical test results concerning the between-task comparison of the two FS processing types on the syntactic level. It shows that the two tasks were strikingly similar in terms of the two broad categories on this level: the category percentages were almost the same across the two tasks. Wilcoxon Signed Ranks tests showed no significant difference in the two broad categories. Despite the overall similarity on the syntactic level, Table 7 reveals a more complex picture: for internal FSs, the learners made significantly less modifications to them in the familiar-topic writing (Percentage, *Z* = −2.193, *p* < 0.05), while the reverse was detected for assembled FSs: the learners made significantly less modifications to them in the unfamiliar-topic writing (Frequency, *Z* = −2.869, *p* < 0.01; Percentage, *Z* = −2.812, *p* < 0.01).

**Table 6:** Between-task comparison on the syntactic level.

Categories	Frequency				Percentage (%)			
	Familiar	Unfamiliar	*Z*	Sig.	Familiar	Unfamiliar	*Z*	Sig.
Intact integration	29.8 (6.4)	24.7 (3.6)	−1.790	0.074	67.33 (11.10)	65.73 (9.47)	−0.614	0.539
syntactic modification	15.3 (7.8)	13.1 (4.3)	−1.131	0.258	32.67 (11.10)	34.27 (9.47)	−0.614	0.539

Familiar, Familiar-topic writing; Unfamiliar, Unfamiliar-topic writing; Standard deviation is given in parentheses.

**Table 7:** Between-task comparison within internal FSs and assembled FSs on the syntactic level.

Categories		Frequency				Percentage (%)			
		Familiar	Unfamiliar	Z	Sig.	Familiar	Unfamiliar	Z	Sig.
Internal FSs	Intact integration	22.9 (6.6)	16.2 (3.3)	−2.710	0.007**	50.54 (6.46)	43.04 (8.45)	−2.298	0.022*
	Syntactic modification	11.6 (6.2)	11.9 (4.7)	−0.358	0.720	24.63 (10.23)	30.94 (10.77)	−2.193	0.028*
Assembled FSs	Intact integration	6.9 (3.1)	8.5 (2.8)	−1.611	0.107	16.80 (9.43)	22.69 (8.11)	−1.990	0.047*
	Syntactic modification	3.7 (2.3)	1.2 (0.9)	−2.869	0.004**	8.04 (3.78)	3.33 (2.75)	−2.812	0.005**

Familiar, Familiar-topic writing; Unfamiliar, Unfamiliar-topic writing; Standard deviation is given in parentheses. *, significant at *p* < 0.05; **, significant at *p* < 0.01.

Furthermore, on the lexical level, the proportion of holistic processing was 71.12% in the familiar-topic writing and 68.08% in the unfamiliar-topic writing. Such high similarity resulted from the fact that the percentage of internal FSs was about the same in the two tasks. On the syntactic level, the proportion of holistic processing was 50.54% in the familiar-topic writing and 43.04% in the unfamiliar-topic writing. This difference can be attributed to the fact that learners had more intact integration of internal FSs in the familiar-topic writing. Taken together, the results showed that topic familiarity may lead to higher proportion of holistic processing on the syntactic level, but not the lexical level.

## 5 Discussion

### 5.1 L2 learners’ productive processing of FSs

In line with the first hypothesis, the learner’s FS processing types can be categorized on the lexical and syntactic levels. Furthermore, the findings partially support the first hypothesis in that there was more holistic processing than compositional processing on the lexical level, but not on the syntactic level. Specifically, the high proportion of holistic processing on the lexical level was mainly driven by the frequent retrieval of internal FSs, while the reduced proportion of holistic processing on the syntactic level was mainly caused by the substantial amount of modification made to the internal FSs.

#### 5.1.1 FS processing on the lexical level: frequent retrieval of internal FSs

On the lexical level, the retrieval of internal FSs had a frequency of 35.75, accounting for 76.68% of FS processing. This result indicates that the learners frequently retrieved internal FSs from their mental lexicon, given the short length of the writing (about 200 words). This contradicts Wray’s (2002) claim that classroom L2 learners tend to store words separately, but supports other previous findings that learners retain information about the co-occurrence of words. Wray (2002) claimed that the creation of L2 lexicon is fundamentally different from that of L1 lexicon. When encountering *major catastrophe*, native speakers would notice and store it as a sequence. In contrast, L2 learners would analyze it into individual words. Consequently, they are likely to acquire a lexicon consisting of single words. However, counterevidence has been found against this claim. For example, repetition can promote the incidental learning of L2 collocations (e.g., Durrant and Schmitt, 2010; Webb et al., 2013); L2 learners are sensitive to the phrasal frequency of FSs (e.g., Ellis et al., 2008; Wolter and Gyllstad, 2013; Wolter and Yamashita, 2018; Northbrook and Conklin, 2019; Öksüz et al., 2020). Along these lines, the current findings suggest that L2 learners have considerable storages of FSs in their mental lexicon.

It can be argued that even though learners may tend to break down word sequences into individual words, they may also pay attention to how the words glue together and memorize the sequences as wholes. In other words, they may attend to individual words and the whole FS simultaneously. Furthermore, learners’ formation analysis of FSs could actually facilitate retention, as seen from the interview excerpt:

(be faced with) This form felt a little strange. It could express a sense of enforcement, as if the fate compels you to face it, like “face somebody something.” You are compelled to face it, rather than voluntarily. (HS5)

In this example, the student analyzed the semantic structure of the FS to understand the form-meaning mapping (the reason why the form expresses the meaning), which could potentially help memorization. Actually, the mnemonic benefits of learners’ analysis of FSs have been increasingly recognized and verified (e.g., Boers and Lindstromberg, 2009; Hatami, 2015).

#### 5.1.2 FS processing on the syntactic level: substantial amount of modification

On the syntactic level, the result that syntactic modification accounted for 33.47% indicates that a substantial proportion of FSs were indeed modified at the time of use. This result converges with previous findings that point to the syntactic flexibility of FSs (e.g., Gibbs and Nayak, 1989; Barkema, 1994; Moon, 1998; Grant, 2003; Wulff, 2008; Kyriacou et al., 2020). For example, Wulff (2008) found that the majority of the targeted idiomatic V NP-constructions did not deviate strongly from the baseline in terms of syntactic flexibility. Kyriacou et al. (2020) showed that nontransparent idioms can be passivized while retaining their figurative meaning. These previous findings point to the fact that FSs are much more flexible than commonly assumed, and the current findings testify to this from a process-based, learner-internal perspective.

Consequently, the implication is that the corpus-driven approaches which did not take syntactic flexibility into account might overlook many genuine instances of FSs and thus seriously underestimate the frequency of flexible FSs (e.g., Biber et al., 1999; Staples et al., 2013; Huang, 2015). For example, Biber et al. (1999) noted that verb phrase bundles were rarely found in academic discourse. Despite register influence, such rarity is possibly because verbs in English are most likely to occur in various, inflected forms which might be missed by the concordancers. By comparison, corpus-driven approaches that used lemmatized frequency counts and allowed distances within collocations can better accommodate the syntactic flexibility of FSs (e.g., Vincent, 2013; Durrant, 2014; Yoon, 2016).

Additionally, what is particularly noteworthy about learners’ syntactic modification of FSs is that such modification might lead to errors in FS use. It was found that students might retrieve a correct FS from the mental lexicon, but made inappropriate modification to it. For example, a student modified *suffer from* inappropriately:

They *are usually suffered from the academic stress.

I changed “suffer from” into passive form. They are tortured by something. These victims should be passive, being scared. (LS3-Familiar)

In this example, an error occurred as the student did not master the usage of the FS adequately. This shows that knowing the basic form of an FS is just the first step, while being able to integrate it appropriately into writing is equally important.

#### 5.1.3 A model of L2 FS production

**Figure 4:**
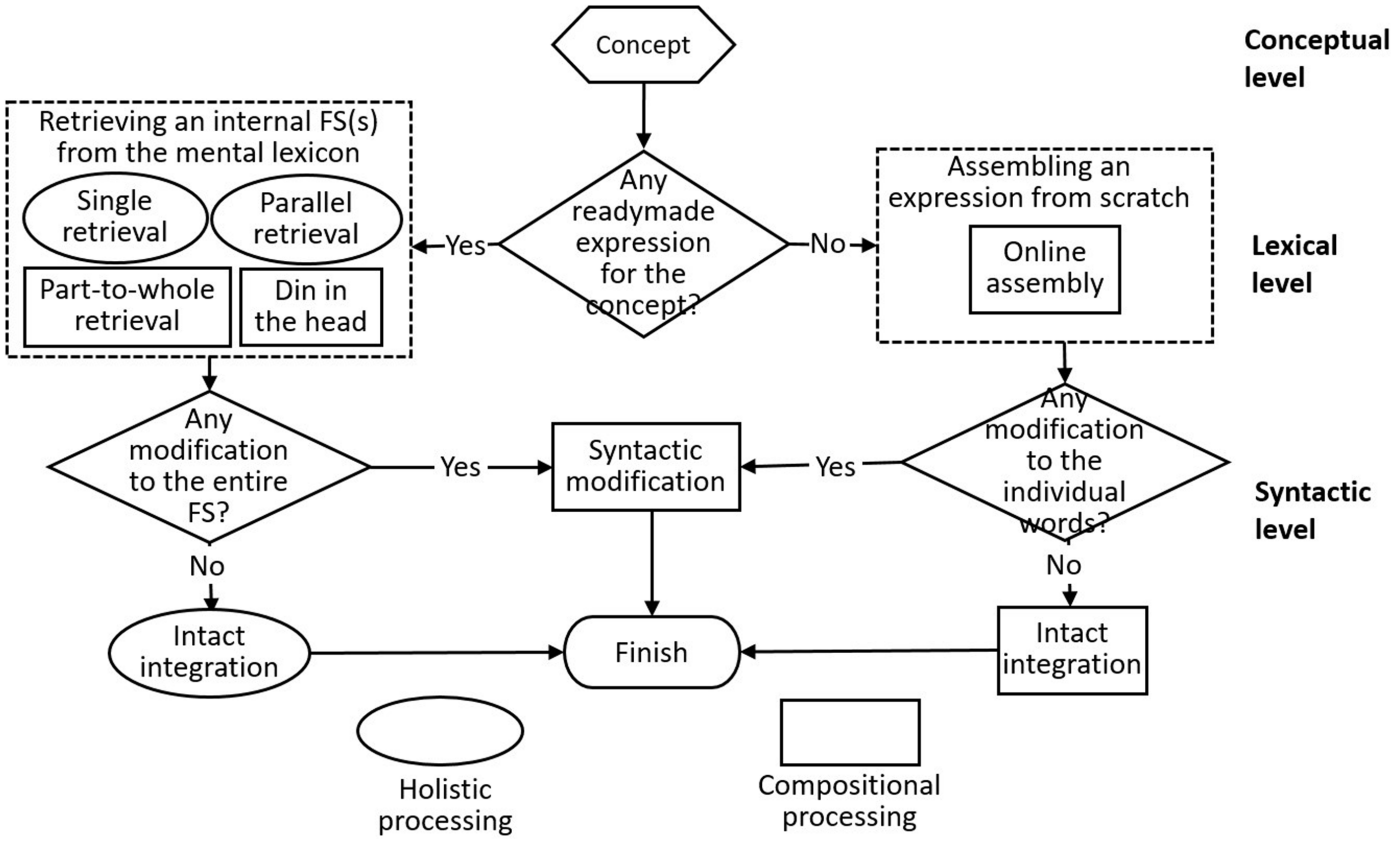
A model of L2 formulaic sequence production.

Based on previous hybrid models of idiom production (e.g., Cutting and Bock, 1997; Sprenger et al., 2006) and the current categorization of FS processing types, a model of L2 FS production was proposed (see Figure 4). This model consists of three processing levels: conceptual, lexical and syntactic. After generating a concept, the learner either retrieves an internal FS (s) from the mental lexicon (which can take the form of single retrieval, parallel retrieval, part-to-whole retrieval and “din in the head”), or assembles an expression from scratch (online assembly). Subsequently, the learner embeds the retrieved or assembled FS in the text through either intact integration or syntactic modification. Concerning the characteristics of the processing types, the ellipse in the figure indicates holistic processing, while the rectangle indicates compositional processing.

By specifying L2 learners’ FS processing types on different levels, this tentative model of L2 FS production extends previous hybrid models of idiom production, thus contributing to the “holistic or compositional” debate on FS processing. The previous hybrid models were proposed for L1 idiom production, which is supposed to be highly automatized. However, the production of L2 FSs might be a more complex and effortful process, as Jiang (2000) contended that the development of most L2 words would fossilize before reaching the final, native-like stage. In the current model, on the lexical level, part-to-whole retrieval, “din in the head” and online assembly all entail some constructional efforts. For L1 speakers, the FS basic forms are supposed to be retrieved holistically due to deeply entrenched mental representations. However, for L2 learners, their mental representations of even internal FSs may be relatively weak, which is manifested by part-to-whole retrieval and “din in the head.” Besides, they do not have unitary representations of the assembled FSs (at least not perceivable), so they have to construct these FSs from single words. On the syntactic level, syntactic modification is proposed as an FS processing type. According to Jiang (2000), whereas the production of morphologically appropriate words tends to be an automatic process for L1 speakers, it is likely to be “a conscious process involving two strictly serial steps: the selection of a root form such as *leave* and then the selection of the morphologically appropriate form” (p. 58). In the current model, these two serial steps occur on the lexical level and the syntactic level, respectively. Therefore, while previous hybrid models only indicate that idioms can be compositional, without denoting any consciousness of modification on the speakers’ part, this study establishes syntactic modification as a conscious manipulation by L2 learners.

By elaborating the partial compositionality of FSs on different processing levels, this study can help to interpret the inconsistency between previous findings. Using computer keystroke recordings, Yuan and Xu (2016) found that controlled processing of FSs was most frequent, which denotes dis-fluent production. According to the current model, such dis-fluency might result from the compositional processing on the lexical and syntactic levels. Actually, the percentage of “single/parallel retrieval → intact integration,” entailing holistic processing on both levels, was 43.67%, which is strikingly similar to the result of Yuan and Xu (2016) that automatic processing only accounted for 41.98%. By contrast, using think-aloud, Xu (2010) found that automatic retrieval dominates. This might result from the high percentage of fluent processing types on the lexical level (the accumulated percentage of single/parallel retrieval, fluent part-to-whole retrieval and fluent online assembly was 90.37% in the current study), since think-aloud method is capable of revealing the learners’ mental processes on this level.

### 5.2 Effects of L2 proficiency on learners’ productive processing of FSs

The findings partially support the second hypothesis in that higher L2 proficiency led to more holistic processing on the lexical but not the syntactic level. Specifically, compared with lower-proficiency students, higher-proficiency students had retrieved more internal FSs and made more syntactic modifications to them.

Higher-proficiency students retrieved more internal FSs probably because they had higher awareness of FS memorization and thus larger FS storage. In the interview, when asked about whether they would intentionally collect and memorize FSs in normal situations, all the higher-proficiency students responded affirmatively, while three out the five lower-proficiency students responded negatively. With higher awareness of FS memorization, higher-proficiency students would not only fulfill conscientiously the FS memorization tasks assigned by their teachers, but also pay attention to the worthy FSs on their own initiative, as seen in the following interview excerpt:

Our teacher would note the phrases for us. I would mark them with the highlighter, so they would look like chunks. During morning reading, I would recite them. When reading passages, I myself would note down the phrases that seemed good and valuable. (HS3)

Contrastively, with lower awareness of FS memorization, lower-proficiency students tended to only passively memorize the FSs under teachers’ requirement, as seen in the following interview excerpt:

Our teacher would teach some key phrases. However, to recite or not, was up to us. For me who did not like reciting, I would glance at them over and over, cherishing every moment before the dictation. Then, shut the book and write. (LS2)

Briefly, higher-proficiency students were more like active accumulators of FS knowledge, while their lower-proficiency counterparts seemed like passive receivers in learning FSs. Therefore, the former would have richer FS storage, thus retrieving more internal FSs during writing. This finding is consistent with Chen (2019) result that the high-performance group used more recited collocations than the low-performance group. The author posited that the high-performance learners had developed the habit of reciting collocations as wholes, while the low-performance learners had much lower awareness of collocations. Both studies have underscored high-proficiency students’ high awareness of FS memorization.

The reason why higher-proficiency students made more syntactic modifications to internal FSs may be explained as follows. First, those students probably used more formally flexible FSs such as phrasal verbs which were prone to occur with inflections and additions of modifying elements such as *have caused serious damage to*. By contrast, lower-proficiency students probably used a higher proportion of inflexible FSs, such as *of course*, *at the same time* and *the details are as follows*. It is also possible that they were more likely to store and retrieve fully lexically specified sequences (e.g., *from my perspective*), although these sequences can be seen as instantiations of flexible formulaic frames (e.g., *from…perspective*).

Second, higher-proficiency students might pay more attention to the contextual appropriateness of their FS use such as non-redundancy and grammaticality, so they would tailor the FSs more meticulously for the specific context by making more syntactic modifications. Lower-proficiency students, on the other hand, might sometimes lack consideration about the contextual appropriateness of FSs, thus embedding them directly in the text without proper modification. Similarly, Tavakoli and Uchihara (2020) noted that, more advanced learners could recycle FSs from task prompts competently by using them creatively in various forms, whereas lower-proficiency speakers used them repeatedly in the same form. Both studies have indicated the improved ability of adjusting the FSs to the specific contexts with increased L2 proficiency.

Third, higher-proficiency students might have better understandings of syntactic structures of the FSs, so they could manipulate them more flexibly and make more complex modifications. By contrast, lower-proficiency students might have inadequate understanding of the FS structures, so they could only use the FSs in a rigid way. For example:

This expression (As far as I’m concerned) came out directly. I use it frequently. And there will be no worry about lexical error, because it is a fixed phrase. How nice! (LS2-2)

Acquisition experience:

Our teacher marked out this expression and asked us to memorize it. Actually, I was wondering why adding these words up can mean就我而言, but it does mean this.

As can be seen, due to inadequate understanding of the FS structure, the student would not fully parse the FS and make corresponding modifications to use it in a wider range of contexts.

The observed pattern that higher L2 proficiency led to more holistic processing on the lexical but not the syntactic level echoes the previous findings that L2 proficiency might not affect learners’ FS processing in some aspects (Sonbul, 2015; Yeldham, 2022). However, this result only partially accords with the tendency discerned in Wolter and Yamashita (2018): the degree of holistic processing would increase with the development of L2 proficiency, as higher-proficiency learners showed more reliance on phrasal frequency than lower-proficiency learners.

Two possible reasons can account for such inconsistency. First, the judgment task with decontextualized test items in the previous study might only require minimal processing effort on the syntactic level, so the learners’ performance seemed more reflective of the processing on the lexical level. Contrastively, the writing task in the current study demanded more processing effort on the syntactic level, i.e., embedding the FSs in the text, so it could be quite revealing of the learners’ processing behaviors on this level. Second, the judgment task was administered in a controlled, time-pressured condition, so the learners might prioritize the processing on the lexical level, since it pertains mostly to meaning comprehension (Van Patten, 1990). Contrastively, the writing task in the current research was much more lenient in time. Thus, the learners, especially those of higher-proficiency, would be more consciously engaged in syntactic analysis of the FSs.

### 5.3 Effects of topic familiarity on learners’ productive processing of FSs

The findings partially support the third hypothesis in that higher topic familiarity did not lead to more holistic processing on the lexical level, and it led to more holistic processing on the syntactic level. Specifically, in the familiar-topic writing, although the learners had retrieved more internal FSs in number, the proportion of internal FSs remained stable across the two tasks owing to the parallel increase of assembled FSs in number. Additionally, in the familiar-topic writing the learners made less modifications to the internal FSs.

The result that the learners had retrieved more internal FSs for the familiar topic can be explained from two perspectives: vocabulary readiness (abundant storage of FSs in the mental lexicon) and greater attention to form (more thorough search of the mental lexicon). First, the learners probably stored more FSs related to the familiar content domain. Previous research has shown that topic familiarity can bring about readiness in terms of content and vocabulary (Bui, 2014; Yang and Kim, 2020). Importantly, vocabulary readiness should be reflected in a rich storage of FSs semantically related to the familiar topic. Indeed, as recurrent FSs emerge in and thus depend on specific contexts (Mac Whinney, 2001), researchers have assumed that language users “master formulaic sequences associated with ‘common’ situations better than those occurring in unfamiliar situations” (Forsberg and Fant, 2010, p. 49).

Second, another benefit brought by topic familiarity seemed to be the greater amount of available attention to forms. According to Skehan’s (1998) Limited Capacity Hypothesis, familiar tasks are less cognitively demanding, thus sparing more attentional resources for focus on form. Empirically, previous studies have found that familiar topics led to writing performance with higher lexical complexity (e.g., He and Shi, 2012; Yang and Kim, 2020). Indeed, we found that in the familiar-topic writing, students had made more attempts to upgrade their forms of expressions. For example, the student replaced *all kinds of* with *all sorts of* in his mind:

To express 各种各样, I thought of the simple expression “all kinds of” initially. Then I felt “all sorts of” was more advanced. (HS5-Familiar)

Conceivably, in the familiar-topic writing, the learners would be more capable of attending to form and made more search for advanced expressions, even though the initial expressions were already workable. Thus, more stored FSs would be retrieved.

Nevertheless, the proportion of internal FSs remained stable across the two tasks, since the familiar-topic writing also involved more assembled FSs, which can be further explained by vocabulary readiness brought by topic familiarity as well. For the familiar topic, the learners might have stored more topic-related words, and also be relatively familiar with these words. Therefore, they seemed to have a higher chance of arriving at an acceptable expression through word-by-word assembly. Contrastively, in the unfamiliar-topic writing, without ready-made chunks, they had to frequently abandon the intended meaning or resort to non-formulaic language.

The fact that in the familiar-topic writing the learners made less modifications to the internal FSs might be explained as follows. For the familiar topic, the stored internal FSs might need little or no modification to be integrated into the current contexts, which might be highly consistent with their pervious contexts of use, as they probably had been used repeatedly in the same form to express certain familiar concepts, such as the use of the FS *it is universally acknowledged that* (HS5) as an introducer of familiar phenomena. By contrast, for the unfamiliar topic, the stored FSs may need more modifications to fit the unfamiliar contexts, which may differ considerably from their pervious contexts of use.

Nevertheless, concerning assembled FSs, a reverse situation was found: the learners made less modifications to them in the unfamiliar-topic writing. This is probably because the unfamiliar topic gave rise to more assembled FSs that denote abstract entities and thus do not need inflection in their usage. Specifically, these are the adjective-noun combinations for concepts in the economic field, such as *economic globalization*, *international cooperation*, *free trade*, and *the national economy*. Contrastively, in the familiar-topic writing, the assembled FSs for theme-related concepts tend to denote physical entities or behaviors, and thus occur with inflections. These include phrases describing students’ daily life in explaining causes for stress (e.g., *social activities*, *complex issues*, *intellectual abilities*) and phrases about coping with stress (e.g., *playing games*, *playing sports*, *changing our attitude*). Briefly, concerning assembled FSs, the percentage of intact integration seemed to depend on how many of them denoted abstract concepts, which in turn was arguably determined by the abstractness of the topic, not necessarily the degree of familiarity. However, it might be assumed that the more abstract the topic, the less familiar the learners would be with it.

The observed pattern that higher topic familiarity led to more holistic processing on the syntactic level can help to explain the positive effect of topic familiarity on oral fluency detected in previous studies (Bui, 2014; Bui and Huang, 2018). Bui (2014) found that topic familiarity enabled learners to speak at a faster rate, with a longer mean length of run and fewer pauses. Bui and Huang (2018) showed that topic familiarity could reduce mid-clause pauses. As explained above, learners probably need not make much modification to internal FSs in the familiar contexts. Hence, their computational workloads would be reduced during the familiar-topic task, writing and speaking alike. Therefore, it is reasonable to assume that learners might make less online modifications to internal FSs in familiar-topic speaking tasks as well, thus promoting fluency.

### 5.4 Summary of major arguments

Regarding the first research question, this study proposed that the learners’ FS processing types can be categorized on two levels: lexical and syntactic. On the lexical level, the learners engaged in the retrieval of internal FSs frequently, indicating that they have sizable storages of FSs. This lends support to previous findings that learners retain information about word co-occurrence. On the syntactic level, the learners engaged in syntactic modification of FSs to a considerable extent. This testifies to the syntactic flexibility of FSs detected in previous studies. Consequently, the learners had more holistic processing than compositional processing on the lexical level, but not on the syntactic level. In addition, a model of L2 FS production was proposed, which depicts the learners’ FS processing types on different levels.

Regarding the second research question, this study proposed that, on the lexical level, higher-proficiency students engaged in the retrieval of internal FSs more frequently owing to their higher awareness of FS memorization and larger FS storage. On the syntactic level, they engaged in syntactic modification of FSs more frequently due to their use of more formally flexible FSs, greater attention to the contextual appropriateness of FSs and better understanding of FS structures. Consequently, higher L2 proficiency led to more holistic processing on the lexical but not the syntactic level.

Regarding the third research question, this study proposed that, on the lexical level, in the familiar-topic writing, learners engaged in the retrieval of internal FSs more frequently owing to the benefits of vocabulary readiness and greater attention to form brought by topic familiarity; however, they also engaged in more assembly of assembled FS, rendering the proportion of internal FSs unchanged. On the syntactic level, in the familiar-topic writing, learners engaged in less syntactic modification of internal FSs due to the presumably high consistency between the current and previous contexts of use for those FSs. Consequently, higher topic familiarity led to more holistic processing on the syntactic level but not the lexical level.

## 6 Conclusion

This study investigated L2 learners’ processing of FSs in writing and the effects of L2 proficiency levels and topic familiarity on it. The learners’ conscious processing (retrieval/assembly and integration into the text) of FSs was categorized on the lexical and syntactic levels, and these processing types were characterized as holistic processing or compositional processing. Results reveal that the learners retrieved FSs far more frequently than they assembled them, and made modification to about one thirds of the FSs. Furthermore, higher-proficiency students retrieved more internal FSs and made more modifications to them than their lower-proficiency counterparts; when writing on the familiar topic, the learners retrieved more internal FSs and had more intact integration of them.

Theoretically, this study bolsters our understanding of the cognitive processes involved in L2 FS production and contributes to the “holistic or compositional” debate on FS processing. Methodologically, it took full account of learner-internal FSs by training and inviting the participants to identify FSs. The training material can serve as a reference for future studies of learner-internal FSs.

Pedagogically, the two-layered categorization of FS processing types could help teachers better understand the causes of error in students’ FS use, so they could prepare preventive measures in a more informed way. On the lexical level, students might combine words inappropriately, or retrieve an erroneous FS form due to memory lapse or incorrect fusion. On the syntactic level, they might make incorrect modifications to FSs or apply FSs too rigidly without contextual considerations. Besides, since FSs can be both holistic and compositional in production, teachers are advised to direct their students’ attention to both the conventionality and flexibility of FSs. While emphasizing the importance of memorizing FSs as wholes, they can expose students to the contextualized uses of formally flexible FSs, and provide opportunities for them to use these FSs with different variations. Additionally, teachers can encourage students to memorize frequently-used, specified forms of some formulaic frames in order to reduce the computational workload during FS production.

Despite its contributions, this study suffered a number of limitations. The first was the small sample size. Future studies could recruit more participants from more different proficiency levels, or with different cognitive styles, to investigate the effects of learner-related factors on FS processing. Second, for the identification of learner-internal FSs, this study relied on the subjective judgments of the learners who might neglect some FSs due to lack of identification experience. Therefore, besides manual identification, further research could gather multiple compositions of the same learner and use concordance tools to extract the frequent sequences as a reference. In this way, FSs acquired more implicitly and thus easily neglected by the learners can be spotted. Third, this study used the method of VSR to investigate the learners’ mental processes in FS production. Although VSRs were conducted immediately after the writing task, some details would inevitably be missed in the recall. Further research is suggested to examine the effectiveness of using VSR and think-aloud in combination. Finally, this study made a distinction between two processing levels to portray the FS processing types. However, the real cognitive processes in FS production might be more complex and defy such simple distinction. The two-layered categorization awaits further verification.

## Data availability statement

The original contributions presented in the study are included in the article/Supplementary material, further inquiries can be directed to the corresponding author.

## Ethics statement

The studies involving humans were approved by Science and Technology Research Ethics Committee/Social Science Sub-Committees of Nanjing University. The studies were conducted in accordance with the local legislation and institutional requirements. The participants provided their written informed consent to participate in this study.

## Author contributions

KF: Writing – original draft, Writing – review & editing, Conceptualization, Investigation. HW: Supervision, Writing – review & editing.

## Footnotes

^1^ https://www.techsmith.com/download/camtasia/

^2^ https://www.iflyrec.com/

^3^We found that fusion accounted for 4%, e.g., *cast a significant influence on* (HS3: Perhaps this was created by myself. I learned that “cast” means 投掷, like in “cast shadow.” I thought it might have a more abstract meaning, so I used “cast a significant influence on.” I have used it frequently in writing since high school).

^4^ https://www.ldoceonline.com/

^5^ http://www.freecollocation.com/
