# Supplementary material for: The productive processing of formulaic sequences by second language learners in writing
Source: Front Psychol. 2024 Feb 21;15:1281926. doi: 10.3389/fpsyg.2024.1281926 (PMC10914984; doi:10.3389/fpsyg.2024.1281926)
Supplement: Supplementary file 1 [file Table_1.docx]

Supplementary Material

# 1. The presentation as the training material of FS identification (This material had been tested and refined by a pilot study)

**语块的定义 [Definition of chunks]**

语块，即块状的语言单位，由多个单词组成，通常被当作一个整体使用，形式固定或半固定，具有约定俗成的特性，如搭配、习语、谚语、惯用语、口头禅等。[Chunks are multi-word units commonly used as wholes. They are conventionalized expressions with fixed or semi-fixed forms. Chunks include such as collocations, idioms, proverbs, routines and pet phrases.]

*a piece of cake*

*be all ears*

*turn a blind eye to*

*take a picture lose weight heavy rain*

*How are you?*

*Help yourself*

*Look before you leap*

*Practice makes perfect*

**语块的分类 [Categorization of chunks]**

| 固定短语  [Fixed phrases] | *in my opinion, generally speaking, as a result*  *lead to, suffer from, pay attention to* |
| --- | --- |
| 惯用小句  [Routines] | *How are you? How do you do?*  *Long time no see.* |
| 短语结构  [Phrasal structures] | *see you… (soon, later, tomorrow…),*  *inform/rob sb of sth,* *provide sb with sth* |
| 常用句型  [Sentence patterns] | *not only…but also… It seems that…*  *There is no doubt that…* |

**语块的筛选 [Identification of chunks]**

至少包含两个单词 [At least two words in length]

以前学过、见过或用过 (自创的后来经常用 也算) [Previously learned, encountered or used, including self-created expressions which were frequently used later]

感觉可以当作一个整体 [Being perceived as a whole]

**The researcher’s explanation of the PowerPoint presentation**

Researcher: Have you heard about the concept of chunk?

Student: Actually, my high school teacher mentioned this before, but I didn’t have time to understand it then. In the university, our teacher hasn’t talked about it in my impression.

Researcher: Alright, let me give you a brief introduction to chunks. Chunks are multi-word units commonly used as wholes. They are conventionalized expressions with fixed or semi-fixed forms. Chunks include such as collocations, idioms, proverbs, routines and pet phrases. Let’s look at some examples of collocations: *take a picture*, *lose weight* and *heavy rain*. We say*拍照*as *take a picture*. We should use *take*, rather than *make* or *pat*, though *pat* means*拍*in Chinese. *Lose weight* means*减肥*. We would use the verb *lose* and the noun *weight*, rather than *reduce fat*. *Heavy rain* means*大雨*, but we would not say*沉重的雨[heavy rain]*in Chinese. We can see that there may be different conventionalized expressions in different languages. Next let’s look at idioms. Idioms have more opaque meanings and more fixed forms, for example, *a piece of cake小菜一碟*, *be all ears全神贯注地听*, *turn a blind eye to熟视无睹*. If we haven’t learned the idioms before, we could hardly guess their meanings. Next, let’s turn to proverbs. Proverbs are similar to idioms, and they usually convey some experience or truth. For example, *look before you leap三思而后行*, *practice makes perfect熟能生巧*. If we haven’t learned the proverbs before, we would not know how to express the meanings. Routines are like the formulas in mathematics that we can apply directly in use. For example, we use *how are you?* for greetings; we use *help yourself* to entertain the guests. The pet phrases of individuals are also chunks. For example, if a person says frequently *don’t get me wrong* or *you know*. These phrases can be seen as chunks to this person.

Next, let’s look at the categorization of chunks. Chunks can be classified into these four types. The first type is fixed phrases, such as *in my opinion*, *generally speaking*, *as a result*, *lead to*, *suffer from*, and *pay attention to*. The second type is routines. They are complete and directly usable sentences such as *how are you? how do you do? long time no see*. The third type is phrasal structures or semi-fixed phrases. For example, *see you...* we can say *see you soon*, *see you later*, *see you tomorrow* etc. We can changes words in its latter part. Other examples are *inform somebody of something*, *rob somebody of something* and *provide somebody with something*. We can fill the slots according to the specific situations. The fourth type is sentence patterns, such as *not only...but also...*, *it seems that...*, *there is no doubt that...*

Next, let’s look at the identification of chunks. There are three identification criteria. “At least two words in length” means that it should include two or more than two words. “Previously learned, encountered or used.” This includes the expressions that were previously created by yourself and later used frequently. “Being perceived as a whole” means that you have some impression of its holistic form in your mind and you do not feel that you assemble it word-by-word on the spot. The above is the introduction to chunks. Is there anything unclear to you?

Student: I have some understanding now. I feel that chunks are similar to “phrases” mentioned frequently in middle school and high school. Chunks include the phrases.

Researcher: That’s right. We can understand chunks as coving a broad scope. Now please identify the chunks in your composition. There are no “right” or “wrong” identifications of chunks, and people may differ in their feelings. As long as you feel that the expression fulfills these three criteria, you can consider it as a chunk. Please mark in red the expressions you considered as chunks. Please mark as many chunks as possible, even if you may not be very sure.

**2. Prompt questions for students’ recall of FS production processes** (The design of these questions was informed by the result of the pilot study, which used think-aloud method. As we found that think-aloud could not reveal details of FS production processes satisfactorily, we used video stimulated recalls in the experiment of the current study.)

1. Did you think of the meaning in Chinese first and then this chunk, or think of the chunk directly? What did you intend to express here?

2. Is this chunk the only expression you came up with? Did you think about other expressions? Did you think out the whole chunk altogether, or a rough image of it first? Did you feel effortful in thinking out the wording?

3. What form appeared in your mind first? Did you make any modification to it according to the context? Did you consider the grammar requirement? Did you come across any spelling problem?

4. Did you make an evaluation of it after you wrote it down? Did you feel satisfied about it? Did you make any revision after the evaluation?

**3. The interview guide of FS acquisition experience**

**Questions about FS acquisition experience in general:**

Please talk about your English learning experience from childhood till now.

1. Did your teachers direct your attention to chunks? How was the English class carried out usually? What classroom activities did you have?

2. Did the textbooks and reference materials direct your attention to chunks? What are the materials that directed your attention to chunks?

3. Did the dictionaries direct your attention to chunks? Would you check the collocations and example sentences when you look up words in a dictionary?

4. Would you memorize the related collocations when you memorize words?

5. Would you collect and memorize chunks intentionally in normal situations? What after-class learning activities did you have?

6. Do you feel that chunks are more difficult to memorize than single words?

7. Do you think chunks are important in learning English? Are they important to writing? How did you practice writing usually? How did you prepare for the national entrance exam? How did you learn the model essays? Would you recite the model essays and the chunks in them?

8. How do you think we can increase our chunk storage? Is there any method that can help memorization?

**Questions about acquisition experience of each FS:**

Please talk about how you learned this chunk. Trying to recall your stories, thoughts and feelings related to it. The more you recall, the better.

1. What is the specific situation that you first encountered it? How did you get a deeper impression of it? How did you met it afterwards?

2. Did you memorize it deliberately? What method did you use to help memorization? For example, memorization with other words or phrases, analogy with similar words or phrases, grouping and organizing lexis, imagination, and memorization with example sentences. Did you think this chunk is easy to remember?

3. Did you understand the meaning of every word in this chunk? Did you think about why this form expresses this meaning? Did you analyze its structure?

4. Have you used this chunk before? What is the specific context of use? Did you make any adjustment to it? Did you use it frequently?

5. Have you ever misused or misunderstood it? Did you have any difficulty in learning it? Please make some comments on it.
