# Supplementary material for: The productive processing of formulaic sequences by second language learners in writing
Source: Front Psychol. 2024 Feb 21;15:1281926. doi: 10.3389/fpsyg.2024.1281926 (PMC10914984; doi:10.3389/fpsyg.2024.1281926)
Supplement: Supplementary file 2 [file Presentation_1.PPTX]

## Slide 1
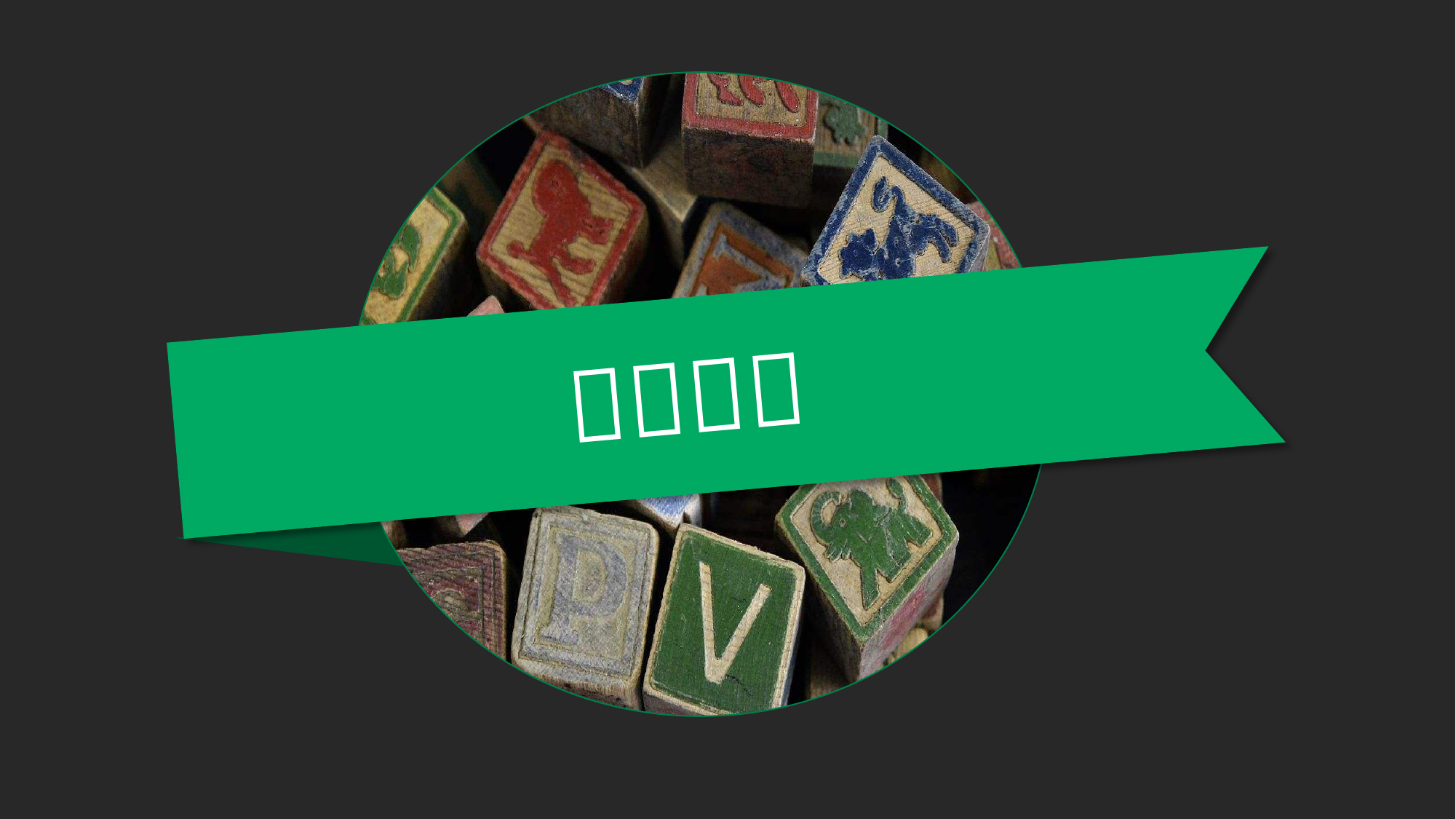

语块简介

## Slide 2
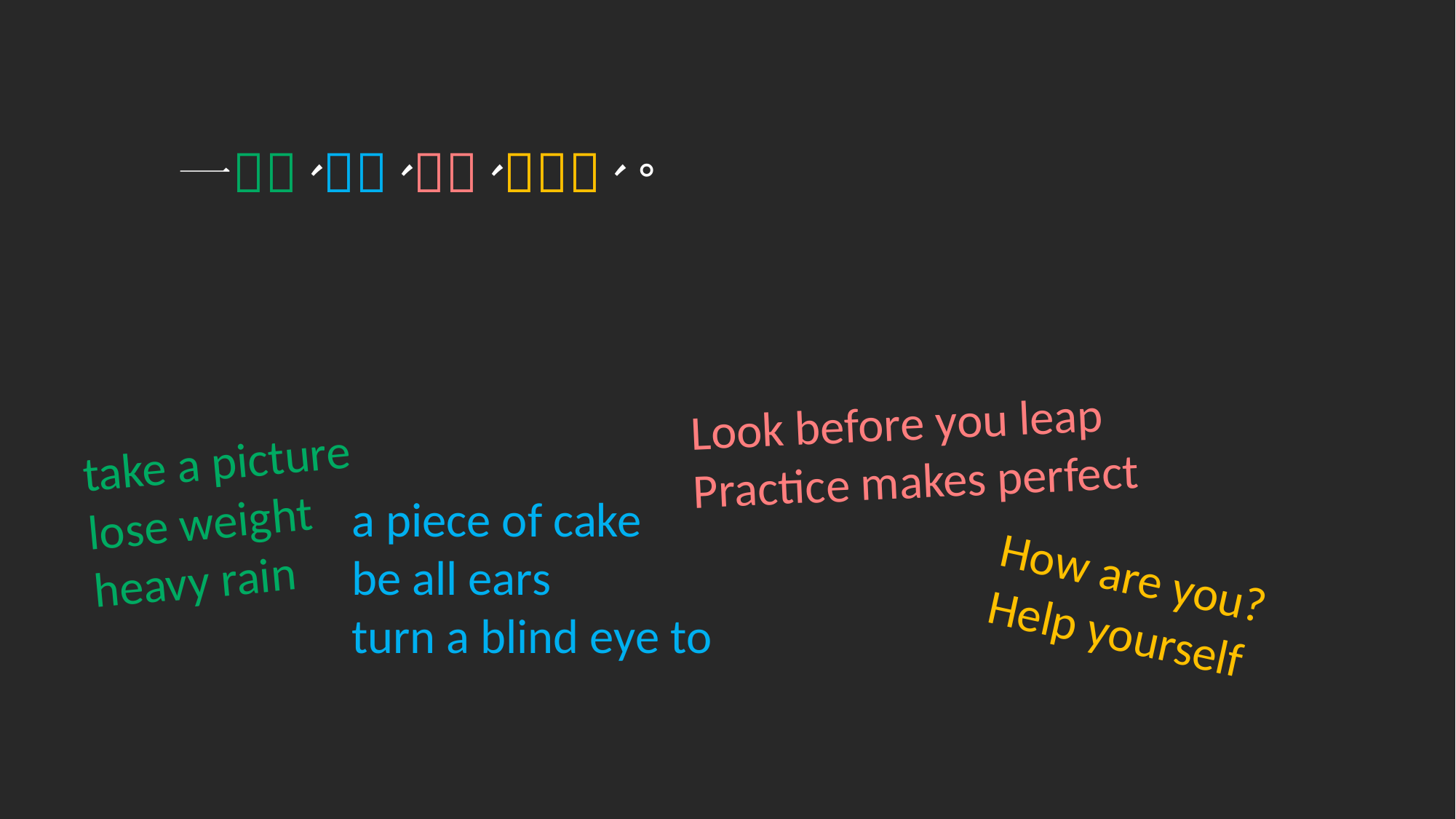

语块，即块状的语言单位，由多个单词组成，通常被当作一个整体使用，形式固定或半固定，具有约定俗成的特性，如搭配、习语、谚语、惯用语、口头禅等。
Look before you leap
Practice makes perfect
take a picture
lose weight
heavy rain
a piece of cake
be all ears
turn a blind eye to
How are you?
Help yourself

## Slide 3
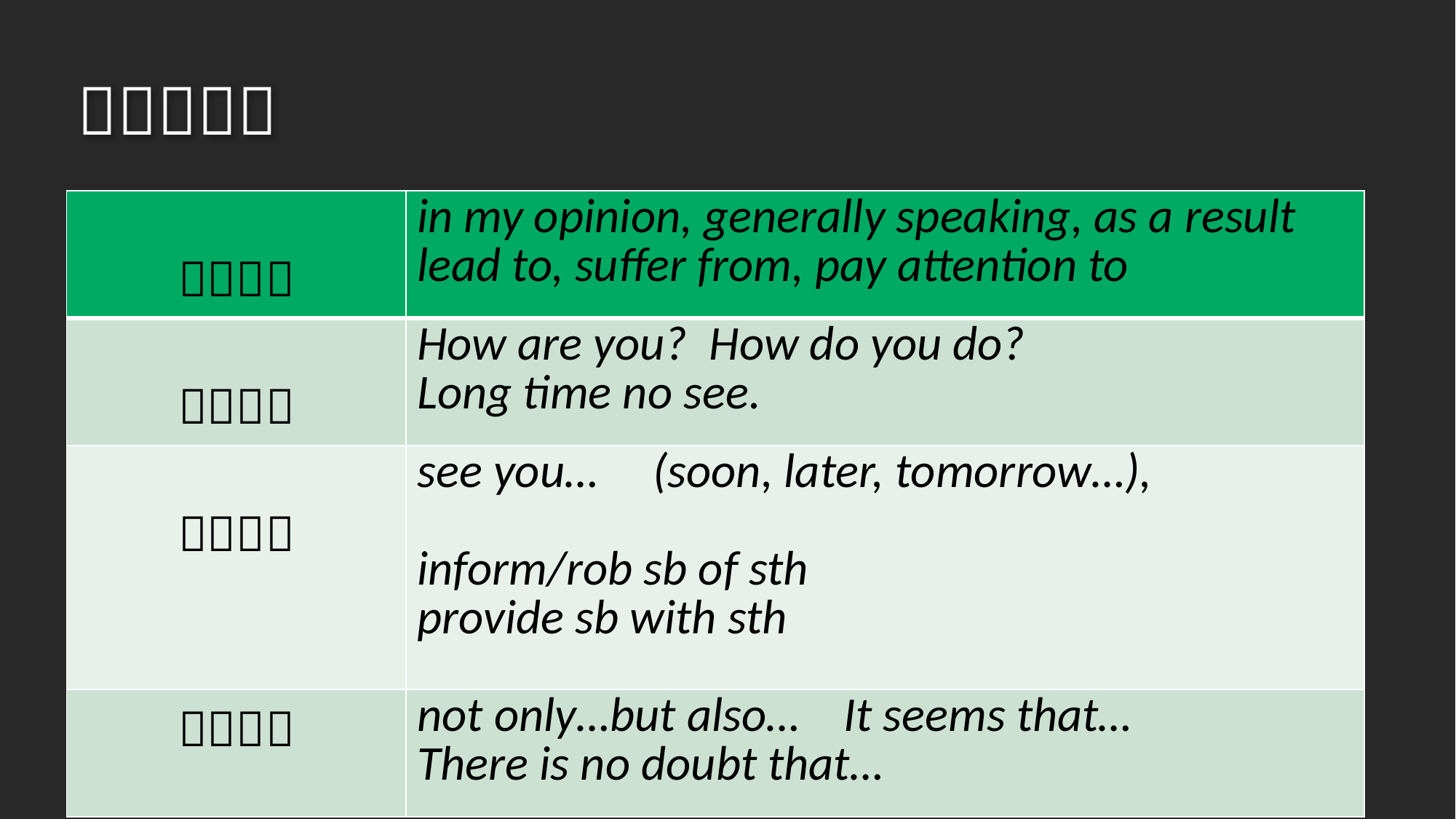

语块的分类
| 固定短语 | in my opinion, generally speaking, as a result lead to, suffer from, pay attention to |
| --- | --- |
| 惯用小句 | How are you? How do you do? Long time no see. |
| 短语结构 | see you… (soon, later, tomorrow…), inform/rob sb of sth provide sb with sth |
| 常用句型 | not only…but also… It seems that… There is no doubt that… |
（）

## Slide 4
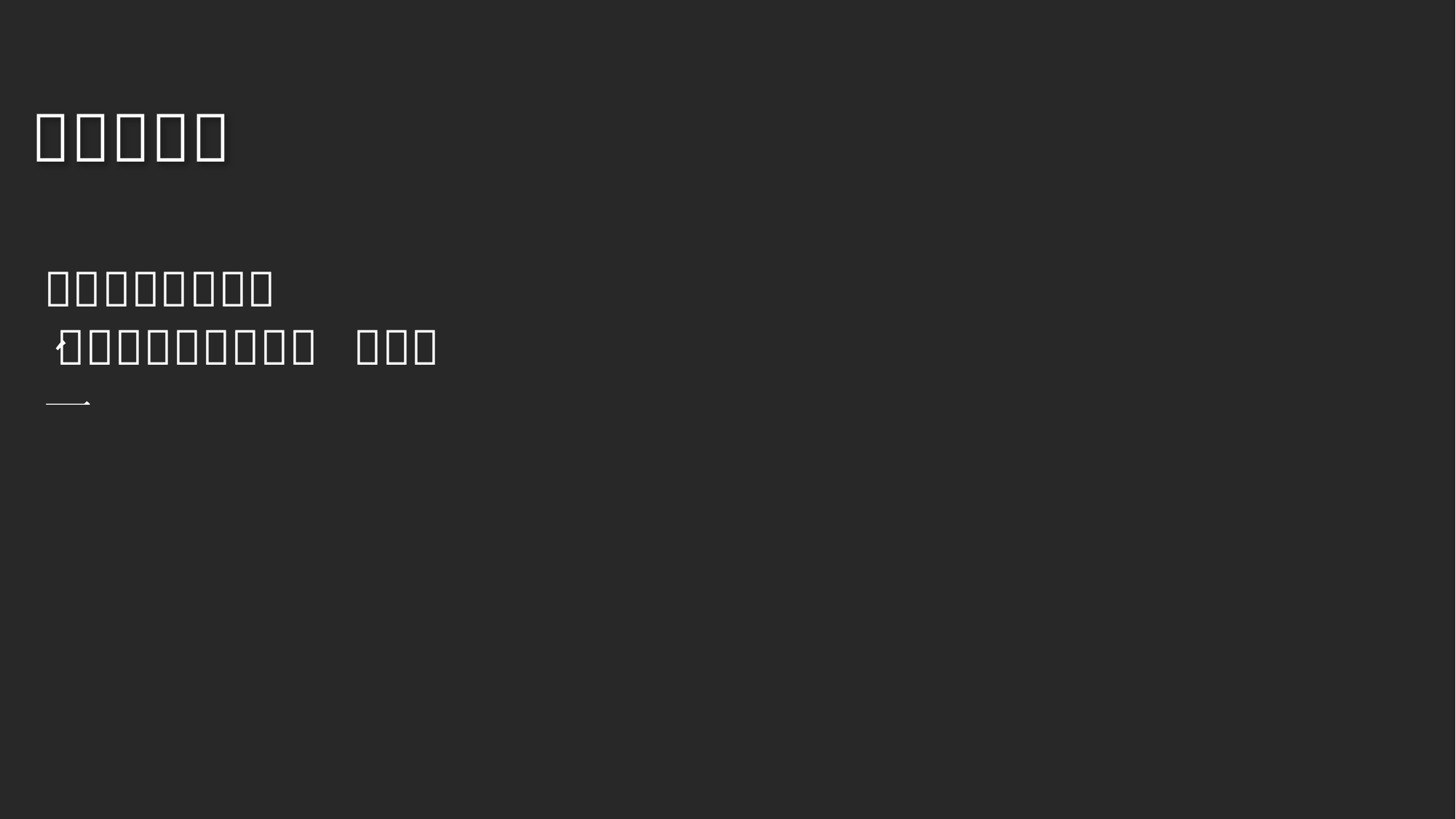

语块的筛选
至少包含两个单词
以前学过、见过或用过 （自创的后来经常用 也算）
感觉可以当作一个整体

## Slide 5
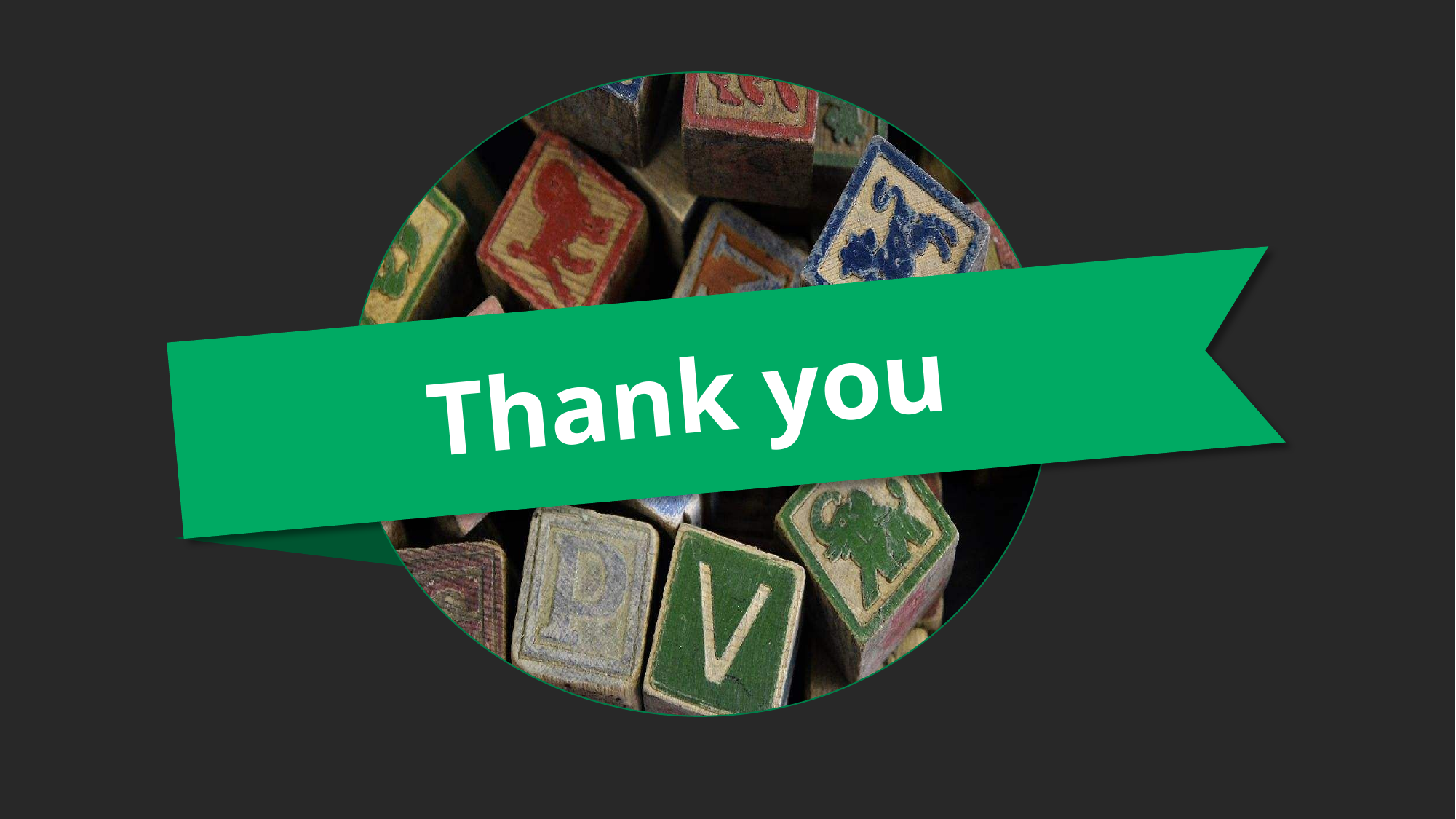

Thank you
